# Supplementary material for: Exploring strategies for management of in-hospital stroke in Sweden: A qualitative study
Source: PLoS One. 2024 Nov 26;19(11):e0313765. doi: 10.1371/journal.pone.0313765 (PMC11594569; doi:10.1371/journal.pone.0313765)
Supplement: S1 Table — (DOCX) [file pone.0313765.s001.docx]

**S1 Table.** Aggregated lead times to thrombolysis administration for IHS and COS patients between 2010 and 2019 at the six included sites.

| Hospital | IHS | | COS | |
| --- | --- | --- | --- | --- |
|  | DNT (min) | ONT (min) | DNT (min) | ONT (min) |
| A | 135 | 135 | 45 | 150 |
| B | 52 | 65 | 27 | 109 |
| C | 59 | 59 | 35 | 110 |
| D | 80 | 120 | 27 | 115 |
| E | 85 | 134 | 48 | 123 |
| F | 46 | 49 | 50 | 129 |

COS: community onset stroke; DNT: door to needle time; IHS: in-hospital stroke; min: minutes; ONT: onset to needle time
