# Supplementary material for: Exploring strategies for management of in-hospital stroke in Sweden: A qualitative study
Source: PLoS One. 2024 Nov 26;19(11):e0313765. doi: 10.1371/journal.pone.0313765 (PMC11594569; doi:10.1371/journal.pone.0313765)
Supplement: S1 Text — (DOCX) [file pone.0313765.s002.docx]

IB: I’ll just go ahead and turn on this recording.

Inf1: Do it, absolutely. So...

IB: And so, [clears throat]. Do you have any questions before we start? I can just briefly explain... You’ve received a document that you’ve probably skimmed through at least briefly.

Inf1: Mm, that was a long time ago, yes. You can explain – mm, absolutely, yes.

IB: So, it’s like this: within my PhD project, we are looking at patients who suffer from a stroke while they are in the hospital. It’s part of a project based on Riksstroke.

Inf1: Mm.

IB: The idea behind this study is to try to look at the routines in Sweden for stroke occurrences in hospitals. [Hospital] was selected as one of the hospitals to participate in the study. The purpose is for you to tell me a bit about how you handle things, how you feel it works, and so on.

Inf1: Yes.

IB: Just to clarify, the information will be handled as confidentially as possible.

Inf1: Mm.

IB: So, if someone has very good insight into stroke care in Sweden, they might be able to figure out that it’s you, but everything will be pseudonymized. If you don’t want to participate after the interview, you can always withdraw your consent.

Inf1: Yes!

IB: Does that sound okay?

Inf1: Yes, that’s fine, mm.

IB: I thought we could start with you briefly telling us who you are, what your professional title is, your specialist training, and how long you’ve been responsible for the stroke alert chain at [hospital].

Inf1: Mmm, okay, eh... Sure, [first name, last name], senior physician in neurology at [hospital]... and... what did you say, how long I’ve been responsible for the stroke alert chain?

IB: Mm.

Inf1: Ehm, [glitch in the recording] I don’t quite remember... maybe... it was since – actually since I became the head physician for the stroke unit, about four or five years ago... first I was co-head physician, and for the last year I’ve been the sole one...

IB: Right...

Inf1: Something like that, mm...

IB: Before this interview, you sent me the protocols for the stroke alert chain, and I can see from it that there’s a routine at [hospital] for how to act if a patient is suspected of having a stroke while admitted to a ward.

Inf1: Mm.

IB: Could you go over what those steps look like?

Inf1: Mm, well, it’s a bit specific because you can have a stroke at [another hospital] or [a third hospital] as well – I don’t know if you count that as inpatient, because it’s not the same hospital grounds...

IB: Mm.

Inf1: I’m not sure what you’re thinking, should I explain...

IB: Let’s focus on [hospital] itself for now.

Inf1: Ah, maybe.

Inf1: Mm... Yes... it’s quite simple, the routine is the same as when we get an alert from anywhere else. You simply call the thrombolysis on-call physician if you’re a doctor in another ward, including the emergency department. The thrombolysis on-call physician handles the alert exactly like any other stroke alert.

IB: Mm.

Inf1: So, we haven’t made it too complicated, we just follow our regular stroke alert chain.

IB: Yeah...

Inf1: The point is to do things as quickly as possible. The doctor who identifies the patient with stroke symptoms calls the emergency phone. Our on-call neurologist or stroke physician answers, and the caller describes the symptoms and when they started. They also try to give an NIHSS score, though they don’t always manage that. The neurologist or stroke physician can quickly assess if the patient is a candidate for thrombolysis or thrombectomy.

IB: Mm.

Inf1: Then, in most cases, we meet at radiology without going to see the patient first, to save time. The patient is taken straight to the CT scanner, and after the first CT, we do a neurological exam and decide if a CT angio is necessary, based on whether there’s enough evidence of a proximal occlusion.

IB: Mm.

Inf1: Many years ago, we used to examine more patients before deciding, but we believe we now catch more cases and save time by taking most patients to the scanner directly.

IB: Do you have any idea when you started going straight to radiology, roughly which year?

Inf1: Ah, no [laughs]. We started doing that when we set up our stroke alert chain, but I feel it’s become more frequent, maybe about eight years ago.

IB: Mm.

Inf1: But...

IB: Sorry...

Inf1: No, it’s fine.

IB: So, to clarify: a patient shows stroke symptoms on a ward, often a nurse or sometimes the ward physician identifies it. Let’s say for the sake of example it’s the ward physician who’s there. Is that person the one who calls the thrombolysis on-call physician?

Inf1: Yes.

IB: Is the thrombolysis on-call physician and the primary neurology on-call physician the same person, or...?

Inf1: Ah, right. During the day, the thrombolysis on-call physician is a stroke specialist tied to the stroke ward. It’s always a stroke specialist unless there’s a staffing gap. After 4 p.m., it’s the neurology on-call physician who handles thrombolysis alerts.

IB: But it’s just one call needed from the person who identifies the patient to the stroke physician?

Inf1: Yes...

IB: And that person doesn’t need to call anyone else afterwards?

Inf1: No, no, the stroke physician then triggers the stroke alert, and everyone meets at radiology.

IB: Right.

Inf1: The only exception is if the neurology on-call physician is inexperienced and needs to consult the stroke backup physician.

IB: Mm.

Inf1: And when the thrombolysis physician triggers a stroke alert, what happens next?

Inf1: Then radiology gets the alert, prepares their lab, no referral is needed, and radiology calls back quickly to get the patient’s ID number.

IB: Mm.

Inf1: So, you call the switchboard, trigger the stroke alert, and provide the patient’s ID, the location, and your role. The alert reaches radiology, and they call back to confirm the ID. They prepare a lab quickly.

IB: Who takes the patient to radiology?

Inf1: If it’s an in-hospital alert, the doctor who identifies the stroke symptoms brings the patient to the CT scanner as fast as possible. They are told to head directly there with a nurse.

IB: Right.

Inf1: Two people usually come with the patient, yes.

IB: And once at radiology, who’s there?

Inf1: The thrombolysis physician, the radiology technician, and the reperfusion nurse, who also gets the alert. Often, there are other people there too, like students.

IB: Right, and when it’s time to make a decision...

Inf1: Mm...

IB: Everyone’s gathered at radiology, it sounds like.

Inf1: Mm, yes, that’s right. Often, the radiologist gets the alert too and is ready to check the images.

IB: Mm.

Inf1: So, radiology technician, radiologist, reperfusion nurse, thrombolysis physician, and the team from the ward are all there.

IB: I think I understand how that works. Ehm...

Inf1: Hm...

IB: Like, from your experience, do you feel that this works in practice? Do people follow the routine?

Inf1: Eh, yes, I think they do. And it’s, I mean, we can have [laughs]... it can even be an ECMO patient with advanced ECMO support, and we still bring them to radiology.

IB: Mm...

Inf1: Everyone usually cooperates and heads to radiology, so it works well... The routine is well-established.

IB: And if you think about it... it’s maybe a difficult question, but in general, would you say that hospital staff, like those in general wards – medicine, surgery, orthopedics – are aware of what to do if they suspect a stroke?

Inf1: Yes, I think so. It’s rare nowadays that we encounter situations where, for example, a patient hasn’t been noticed... I mean, sometimes there’s no clear time of onset, like when a patient is found in the morning, but...

IB: Hm.

Inf1: We don’t usually experience delays due to waiting for the patient to improve before acting. That doesn’t happen as often anymore. I would know if it did since I receive the reports, so I would have been informed.

IB: Right. Do you know if there’s any kind of information given to new staff, like when someone is newly hired at [hospital], especially on a ward? Do they receive information about this routine?

Inf1: Eh, no... I’m not sure about that. On the emergency ward, they do, though.

IB: Hm...

Inf1: All doctors in the emergency department are informed, I think. They have it written down. So, information goes out there because it’s often that you find a patient with diffuse symptoms, and it turns out to be a stroke. I’m not sure how that’s classified...

IB: I can’t say for sure how that’s counted...

Inf1: No, I think they’re counted in the regular stroke chain...

IB: Hm...

Inf1: But they can also suffer a new stroke in the emergency room... I know, for example, that the thoracic department has had information for many years about how to handle stroke patients, and to call the thrombolysis physician...

IB: Hm...

Inf1: I don’t know if that’s the case for all wards, probably not... eh, so...

IB: I think we’ll move on... to another section... How – I’d like to ask how you feel the acute care chain works for this patient group at your hospital?

Inf1: Ah... I’d say it works well [laughs].

IB: It sounds like you think it does, but... could you elaborate on what you think works well?

Inf1: Eh... well, I think it works as fast as it can, actually...

IB: Hm.

Inf1: Of course, everything can always be improved, but I think it’s efficient. And we also train our new doctors... continuously... so that’s maybe a key to success as well. We have... first of all, anyone who’s going to be on call must complete a thrombolysis training session. It’s a two-hour session with different cases on how to handle various stroke scenarios. And before that, they must read through certain protocols. Then, after that, they shadow someone more experienced during the day, 8 to 4, when we get a stroke alert, and we call the person in the emergency ward who hasn’t been on call yet and let them handle the alert while we supervise...

IB: Hm...

Inf1: Eh, so they train before having to handle an alert themselves. It’s not always in-hospital strokes, but it helps them practice the chain... and what to do before they go on call themselves.

IB: Hm...

Inf1: Let’s see [searching for something]... Yes! Was there anything else I wanted to say, or... ah... Yes.

IB: But...

Inf1: Was there anything else you wondered about? [laughs]

IB: Yes, well, I was just thinking... Since you’ve worked with this for a long time, do you see any critical points in the care chain for in-hospital strokes where delays could occur?

Inf1: Well, it’s mainly if the patients aren’t identified in the wards... That’s critical. If no one realizes that the patient has had a stroke, then...

IB: Hm...

Inf1: That’s where a big delay can happen, of course.

IB: Hm...

Inf1: You can always work more on spreading information to all the wards in the hospital. It’s very labor-intensive, especially in a big hospital...

IB: Hm...

Inf1: But at [hospital], quite a few wards and places reach out, asking us to come and talk, so information does get out, but it’s not really structured.

IB: No.

Inf1: No...

IB: But you’re saying there’s demand for information from the wards at [hospital]?

Inf1: Yes...

IB: ******

Inf1: Yes, I think so, yes.

IB: Hm...

Inf1: And we do talk about it sometimes, of course.

IB: Hm. It sounds like you’re saying that more information could help bridge the gap in identifying strokes sooner.

Inf1: Hm...

IB: In the identification phase, right?

Inf1: Yes. ****

IB: Is there any other critical point in the care chain, do you think, where time might be lost? It doesn’t have to be at [hospital], just thinking freely about where things might get delayed...

Inf1: Well, I think time is lost if you spend time going to see the patient first...

IB: Hm...

Inf1: That’s where a lot of time can be lost. You can get very adequate information just by talking to the person who calls, even if it’s a nurse calling. It usually works quite well. Sometimes, it’s nurses calling, and we do just like with ambulances, asking them to go to the patient and do some tests, like raising their arms. Even if they haven’t done NIHSS, it’s not a big problem. You can instruct them, and they’ll do some NIHSS-related tests and give a good idea of the symptoms.

IB: Hm...

Inf1: Of course, if there are very subtle symptoms, we’ll go and examine the patient. If it’s not about thrombolysis, we go see the patient instead. But over the phone, you can get a pretty good sense of the patient’s condition, I’d say.

IB: Hm...

Inf1: By asking follow-up questions...

IB: Do you feel that the time window for thrombolysis is often passed when a stroke is identified?

Inf1: Yes, either the time window is passed, or they’re in a surgical ward, and they’ve already had surgery... or they’re on the cardiology ward and have already received Heparin, Brilique, and Trombyl, so it’s too much...

IB: So there are often contraindications for thrombolysis?

Inf1: Yes, exactly. Thrombectomy is usually what we can do. Mm...

IB: Is there anything in the care chain at [hospital] that you would like to improve?

Inf1: Eh... There are always things to improve. In the regular care chain for in-hospital strokes, if we had all the time in the world, we’d want to go out and inform the wards on what to do when a stroke is suspected. But I feel it works quite well as it is. We have, I don’t know which protocol I sent you, but we have a checklist, and [another hospital] and [third hospital] also have a checklist for emergency situations. What we’ve been emphasizing more and more is to avoid unnecessary tests and do as little as possible before calling us, so you don’t waste time.

IB: Hm.

Inf1: NIHSS is crucial, as well as making sure the patient is ID-marked. Everything else is less important. I think we used to lose a lot of time years ago by doing too many things with the patient before calling.

IB: Right.

Inf1: Hm.

IB: Could we briefly touch on [another hospital] and [third hospital]? I’m fairly certain that when looking at thrombectomy data in Riksstroke, [another hospital] and [third hospital] fall under the same flag as [hospital]...

Inf1: Yes...

IB: So it’s hard to distinguish between them, which affects lead times. Lead times aren’t a major concern for this study’s design, but...

Inf1: Hm...

IB: What would happen if someone had a stroke in the medicine department at [another hospital] or in surgery at [third hospital]?

Inf1: Well, there’s a special routine for the emergency department at [another hospital] and [third hospital]. But even on the ward, they call the thrombolysis physician at [hospital]. It’s well-established since they don’t do thrombolysis at [another hospital] or [third hospital]... so it’s the same procedure...

IB: Hm...

Inf1: The only thing is that we transport the patients to [hospital] for the examination. If it’s not likely to require thrombectomy, we might do a CT angio at [another hospital] or [third hospital]. It happens, but there’s no exact routine for when that happens, usually only for very low suspicion cases.

IB: Mm...

Inf1: Otherwise, it’s the same routine when they have a stroke on the ward.

IB: Mm.

Inf1: Mm.

IB: But there are scenarios where the patient is scanned on-site?

Inf1: Yes...

IB: And they might even get treatment on-site?

Inf1: Eh, no... no thrombolysis is done at [third hospital] or [another hospital].

IB: So if you...

Inf1: If they’re mistaken, say they find an occlusion on a scan at [another hospital] or [third hospital], they never administer treatment. They call an ambulance and the thrombolysis physician at [hospital], and the patient is transferred here for treatment. The drive between hospitals takes about 20 minutes, so...

IB: Hm...

Inf1: They wouldn’t have time to start treatment on-site; it’s quicker to bring the patient here.

IB: Hmm, hmm...

Inf1: We’ve had other hospitals where it takes longer to administer thrombolysis, so we get patients here faster for treatment.

IB: Just for my knowledge of the rest of [region], it’s only [another hospital] and [third hospital] where in-hospital strokes go to [hospital].

Inf1: Yes.

IB: That’s not the case for [fourth hospital] or [fifth hospital]?

Inf1: No, no.

IB: They handle their own in-hospital stroke patients?

Inf1: Yes, unless they find an occlusion...

IB: Mm.

Inf1: Plus, we also serve as their stroke backup consultants in the evenings, from 4 p.m. to 8 a.m. for complex cases or difficult decisions about administering thrombolysis.

IB: Just one last question...

Inf1: Mm.

IB: Have you received any improvement suggestions from the staff in general? For example, you mentioned the demand for stroke identification training. Is there anything else the staff has suggested?

Inf1: Mm, from other wards, you mean?

IB: Yes, from the staff at [hospital] in general – doctors, nurses, assistant nurses.

Inf1: You mean those outside the neurology ward, right? Mm...

IB: It can be from the neurology ward too.

Inf1: Mm... no, I haven’t received many specific improvement suggestions... we do have stroke simulations, both on the simulator center and for thrombectomy. But no... improvement suggestions... I mean, we try to improve continuously, like with the checklist, although it hasn’t been updated in almost a year... Covid added extra routines, but we usually update it about twice a year to make small changes.

IB: Hm...

Inf1: So... in-hospital stroke follows the same regular routine, so it doesn’t complicate things too much.

IB: Mm... well, if there’s nothing else you want to add?

Inf1: No, I’ll just add that since in-hospital strokes are treated exactly the same as other strokes, it’s not complicated. We follow the same routine as when ambulances call about a stroke, so it’s straightforward. The only problem arises if the stroke isn’t identified early.

IB: Hm...

Inf1: But that’s difficult to predict... you can’t always foresee a stroke.

IB: Just one quick question. Once the images are taken and everyone is at radiology, can things get held up at that point?

Inf1: Not during the day, 8 a.m. to 4 p.m. If we’re experienced, we call intervention immediately when we don’t see a bleed. At night, things can take longer with less experienced staff, but there’s always a radiologist on-site.

IB: Hm...

Inf1: And usually, they consult with the stroke backup on the phone. If we see a blockage, intervention is called, and the process moves forward.

IB: So, intervention isn’t on-site at night?

Inf1: No, but they can be there in about 20 minutes once they’re called.

IB: Hmm, hmm...

Inf1: At night, the intervention team needs to be called in. But we follow the same steps: once intervention gives the green light, a thrombectomy alarm is triggered, and the patient is moved to intervention. Anesthesia is already set up when they get there.

IB: Hm...

Inf1: It flows pretty well.

IB: Yes, it sounds like it.

Inf1: The only problem is when we have multiple alarms at the same time...

IB: Right.

Inf1: ...which can happen at night when there’s only one on-call doctor. During the day, we help each other out to avoid delays.

IB: I think that’s all the questions I have.
