# Supplementary material for: Exploring strategies for management of in-hospital stroke in Sweden: A qualitative study
Source: PLoS One. 2024 Nov 26;19(11):e0313765. doi: 10.1371/journal.pone.0313765 (PMC11594569; doi:10.1371/journal.pone.0313765)
Supplement: S2 Text — (DOCX) [file pone.0313765.s003.docx]

IB: Could you briefly tell me what your job title is, your specialist training, and how long you've been responsible for “Rädda Hjärnan” (Save the Brain)?

Inf2: Eh… I am… my pos – was it position, education, and time of responsibility?

IB: Exactly, job title…

Inf2: Yes, specialist physician… eh, and then what was it?

IB: Specialist training…

Inf2: Yes, neurology, exactly, single – single specialist. Ehm, and… I’ve been more or less responsible for “Rädda Hjärnan,” at least coordinating things… so at least coordinating things… like writing protocols and the like. Ehm, I have it all written down [searches on computer]… five years!

IB: Yes.

Inf2: Then I haven’t written everything, and I haven’t done everything, but I’ve been heavily involved since 2016.

IB: Yes! My first question to you then is… is there a routine at your hospital for how to act if an inpatient is suspected of having a stroke?

Inf2: Yes! It depends on what you mean by routine, though, but yes, there is.

IB: Is there a – a written document that all staff have access to?

Inf2: Well – with access to, eh… eh… or like this, eh… it sounds bet – or like this: It’s true if I say yes… but it sounds better than it really is in practice. Eh, because… the document, which is brief, where this actually is written, is hidden in a big document about stroke… itself hidden in a database of documents… and I don’t for a second believe that anyone who’s actually in this situation would find that document useful. So – so, unlike us who work day-to-day in the “Rädda Hjärnan” chain, who find that document very useful – we know where it is, there’s nuanced information in it… “what is the acceptable INR level”... eh, and so on, lots of nuanced details that we find very useful…

IB: Mm.

Inf2: But I don’t think at all that people read it beforehand, know about it, eh, know what to do because they’ve read it, eh, bring it up when it’s time… not at all. So just the fact that there is a written routine… there is, eh… but that doesn’t solve the issue, eh… at all. The relevant issue is… whether other departments, like… cardiology, geriatrics, orthopedics… know what to do, and the fact that there is a written routine doesn’t help them at all.

IB: We could come back to this…

Inf2: Mm.

IB: Ehm…

Inf2: But yes! It exists! [laughs]

IB: So, if it exists then…

Inf2: Mm.

IB: Could you describe… roughly what’s in the routine?

Inf2: Ah… it – it says roughly, that… that, eh… you should – or like this: Eh, the whole idea of the document as it’s set up… is that if you come in via ambulance, this applies, and this, and this. Then there’s a section if you come to the emergency department yourself, then this applies, and if you’re already in the hospital, this applies. But just that… eh, as I said, it’s hidden deep in a very large document that people wouldn’t think to look in. But – but what it essentially says – what it should highlight is essentially: “Contact the neurology on-call physician, and then everything will be sorted” [laughs] – that’s what it should say…

IB: What happens when you contact the neurology on-call physician?

Inf2: Then – well… yes! Eh, it depends on how skilled they are, of course. Eh – eh… how much do you want to involve the backup physician in whether or not an alarm should be triggered? Do you want to see the patient first, or is a verbal report enough? Is it such that you – I was just in one of the meetings where we discussed how these alarms should be handled by the switchboard… is it that you [interrupted by noise] – you handle this over the phone because that’s how the alarm has gone out… ehm, and therefore, you can sort out the logistics quickly, or do you first have to transport yourself to the department where the alarm was raised and then start sorting out the logistics? Eh, has the CT scan been done already, or not? Some don’t do it, but some do, they perform a CT scan, bring the patient back, and then think about contacting neurology. So – so, eh… but as soon as you contact the neurology on-call physician, at least… the sequence of events that must happen will be set in motion, like getting the CT scan done, getting the right competencies involved… radiology gets involved, and [the stroke ward], which handles mixing and administering the treatment in radiology, gets involved. It’s only at that point that there’s a real chance that things will actually happen… Eh, and the neurology on-call physician should be involved in the decision-making, in every way, so the earlier you get them involved in all the steps, the more they can drive those steps forward… ehm, that… that solves the issue then.

IB: It feels like a… relevant follow-up question would be… how do you trigger this alarm chain, and who does that?

Inf2: Mm, there are two ways… or – right now there are two ways, soon there will only be one. But, eh… the natural – what I’ve found over the years [interrupted]… is that the natural thinking for… someone who doesn’t know what they’re doing… like, the cardiologist, orthopedic surgeon, all those who aren’t us [interrupted] who are already in the alarm chain… is that you think you’ve got a stroke, and then you call the switchboard and trigger a “Rädda Hjärnan” alarm… because that’s what you do with a cardiac arrest, that’s what you do with other things. Once the alarm is out, everyone comes, and everything gets sorted. But that’s not how our stroke alarms – “Rädda Hjärnan” alarms – work… our “Rädda Hjärnan” alarms are designed for… the vast majority of alarms that come from ambulances, or at least start in the emergency department… meaning that the “Rädda Hjärnan” alarm itself, the one that goes to the pager… that’s only the least important of the three things that should happen from the emergency department… eh, or least important – it is important, absolutely, but… eh, the emergency department should… call… [the stroke ward] and not just say there’s an alarm but give the estimated arrival time and weight… eh, they should call radiology and give the estimated arrival time… and the patient’s personal number to both of them as well… meaning the things you don’t get in a page, it’s a quality – like a quality report that should come.

IB: Mm.

Inf2: Then they should also call the switchboard, the switchboard presses a button, and a page goes out to a lot of people – including the primary on-call physician – and it just says “Rädda Hjärnan.” What usually happens then, in normal alarms, is that you just call the switchboard and say: “Yes, thank you, I know,” because the backup physician called me five to ten minutes ago and gave me the report I need… to act… That’s how our system is built. So back to the question… when, let’s say cardiology, for example – because that’s what happened today, which is why [laughs] they’re the example I’m using. When they call and say: “Trigger a Rädda Hjärnan alarm,” and it turns out they’re from cardiology, what’s shown on the pager… is “Rädda Hjärnan alarm at cardiology”…

IB: Mm.

Inf2: And what happens then… is that the switchboard sometimes gets a little irritated… once it’s happened, then [interrupted] usually we then make contact, and somewhere during that process, it’s decided that the “real” Rädda Hjärnan alarm should be triggered… meaning that we – we, in the stroke field, actually decide that we’re really going to… eh, gather the nurses, make radiology available, do a CT scan with… thrombolysis or thrombectomy intent… ehm, and it’s only when the neurologist does this that, in a way, the “real” alarm starts… ehm, and… yes, so that’s where – what we’re going to start doing in just a week or so is that the first thing that happens when another department calls… you’re the second person to hear this [laughs] because this hasn’t been spread yet… when another department calls, instead of triggering a Rädda Hjärnan alarm, they’ll speak directly to the neurology on-call physician… so that – because that’s what you want, so if it turns out that a Rädda Hjärnan alarm really should be triggered… well, then it’s done… in the usual way… eh, just a little later.

IB: So if I understand you correctly… up until today, really… there are two alarms being triggered?

Inf2: [interrupted] It can happen that two alarms are triggered.

IB: One alarm is triggered that draws attention to the department…

Inf2: Mm…

IB: And, if I understand correctly, in practice, as the on-call physician, you go to the department and check on the patient?

Inf2: Yes…

IB: … and then you decide to trigger the Rädda Hjärnan alarm, and that’s the alarm that’s usually triggered from an ambulance?

Inf2: Yes, exactly!

IB: Where you trigger, for example, radiology’s availability and…

Inf2: Yes, or like this, eh… eh – no – you trigger the resources you want… it’s easy to think [interrupted] of the Rädda Hjärnan alarm as a… selective entity that’s very specific, but it’s not really like that… eh, I… don’t care at all if a Rädda Hjärnan page goes out or not, I care about the stroke team coming to radiology, and that the radiology lab is available and expecting a Rädda Hjärnan case.

IB: Hm.

Inf2: Whether or not a physical signal goes out or not… is secondary to me… and the waiting times and such, which we… eh, calculate as best we can… eh, for “door-to-needle”… they mess up with the “in-house alarms,” because there the “door” is the stroke onset…

IB: Hm…

Inf2: Logically… eh, and my experience is that, yes, they have a fantastically long door-to-needle time because we don’t have a running start… but they have better onset-to-needle times because they’re already on-site. And it’s the latter that really matters.

IB: Mm…

Inf2: And also because every time… one of the key players is someone who doesn’t know what to do… yes, and never will be good at it because they do it too rarely, like… cardiology in this case… so we can never really get it running smoothly. But! But, back to your question. There are two ways cardiology can act. This was one way, where they do what feels intuitive… they call the switchboard and ask them to trigger a Rädda Hjärnan alarm… which is half-real, so to speak… it goes out to everyone who gets the “Rädda Hjärnan” page, yes… and the right things start to happen… but, the stroke team doesn’t go to radiology and wait there for that page, they wait for better information… for the “real” alarm, so to speak.

IB: Okay, so then I misunderstood you, meaning… radiology and… all the necessary personnel are informed already at the first alarm, but they don’t consider it as important as when an ambulance alerts them?

Inf2: They’re on standby. They don’t expect it to escalate to a real alarm because they don’t trust the competence of the person who triggered it… for very reasonable reasons, because… that’s how it should be. It could be a seizure, it could be anything else… eh, so unlike… unlike the alarms that come from ambulances, where the backup physician is the one who decides to trigger the alarm… or alarms triggered by the emergency department, where the primary on-call neurologist is the one deciding… there’s always competence behind those decisions…

IB: Mm…

Inf2: Whereas here, there never is. But, option two is that they call the primary on-call physician directly, and then you sort things out, and eventually, the primary neurologist decides, “Now we’re going to trigger the Rädda Hjärnan alarm.” Depending on the circumstances, it takes varying amounts of time and requires different levels of consultation and so on… eh, but… then only one alarm is triggered, simply… eh, we… we… when we educate, when we go around a bit – we go around a bit, and so on, we tell people: “Call the primary on-call physician”… and then everything gets sorted [laughs], so to speak, that’s just… because, first of all… it avoids the double-alarm and some of the confusion… eh, but… above all, eh… the primary on-call physician doesn’t want to get a call from a department on the other side of the hospital, and have to go there before they can start working… they want to call and start working while they’re talking…

IB: Got it… Ehm…

Inf2: Or… the sensible primary on-call physician wants that, at least [laughs].

IB: Could you give me… you’ve talked about the cardiologists now, where there was a case today…

Inf2: Hm.

IB: Maybe using that as a starting point, how does the alarm chain function in practice?

Inf2: Eh, chaos.

IB: Can you be a bit more specific?

Inf2: Yes, exactly [laughs]. No – it’s always chaotic – eh… and… eh, half the… the energy goes into figuring out what’s actually been done and what the facts are… eh, is it really a stroke, how much of a stroke is it… eh, how severe are the symptoms… eh, what do we know about the timing… all of that needs to be sorted out from the beginning because the person reporting to the primary on-call physician isn’t ambulance or emergency department staff, who are used to knowing what you need to know. First, you have to figure that out, and then also… what’s been done? Who’s been called? Who’s involved, who isn’t? So it’s always a bit chaotic… but when you’re the first one contacted… then a lot of that gets sorted out just because there’s much less chaos, because the first thing is… “Okay, I’m the first thing happening, alright”… now I know that everything that needs to be done is ahead of us, we don’t need to worry about… did they arrive, did they leave, or not. So the only thing you need to do is be sure enough to start moving the patient toward… toward radiology… then things get sorted – then – then… but you need to make sure the radiology referral gets written, you need to make sure radiology is alerted, and that the stroke team is alerted… and that the backup physician is involved, but those things tend to get sorted out… once you start understanding… the situation you’re in… eh, it [interrupted] is never a problem… eh, or like this… when it’s the ambulance… calling, then as the backup physician – you’re used to trying to get a sense of “is this a Rädda Hjärnan patient or not”…

IB: Hm…

Inf2: But with this, you – you have a certain amount of time, you can talk for a while while the ambulance is on its way… because they’re still some distance from the hospital, so to speak… and the ambulance staff has made this call before… much more often than… than this cardiology… junior doctor, eh, has, which in most cases is their first time ever doing it, for most of them… ehm, so – so how you get the right information is much more – and when it’s the emergency department, which sees strokes fairly often, just knowing what you’re interested in goes much faster… eh, so the fact that the person who had the patient is inexperienced, that’s the whole… it can’t be fixed… and… that’s the whole problem here, it… it’s always a new… person, there… eh, and – and with that, I’ve given up on the idea that this will ever go smoothly. It will always be more or less chaotic… I can never expect the person calling to know what they’re supposed to do… so the first thing we do is try to… g – g – gain control of the situation, and then… then things get sorted…

IB: Yes…

Inf2: And – and to educate on this… is a challenge… mm…

IB: In what way?

Inf2: I – I – no, how are you supposed to get… how are you supposed to get, eh, the temporary doctor who’s in their first day on neurology to feel confident that, “when this happens, it will be chaotic” [laughs] “but then it will get sorted”… it – it’s ehm, that… the calm that I and my other senior colleagues have in this, because we’ve done it so many times that it’s… it’s routine… they don’t have that, so the fact that something will be, and everyone else is new too, as it is in the summer… ehm, that…

IB: Hm…

Inf2: That’s scary – and I can’t fix… that. Because there’s a little detail here too, which we… which we have to focus our education on instead… and that is… ehm, if you come as the primary on-call… in these cases… and, ehm… ehm… and you realize that “no, wait now, we’re going to trigger the alarm here, we’re really going to go for it”… eh, the one thing you absolutely must not do is what feels right, which is to call the switchboard and trigger a Rädda Hjärnan alarm… because then it’s just you who gets paged. Because again, the stroke team and… and radiology, they don’t really trust an alarm that comes from somewhere else, they – they need to be called and given a report, and that’s when they act, because that’s how it’s supposed to be… so the thing you must know… that’s what we emphasize… when we educate on in-house strokes… is just that, eh, when… you, when you actually want the alarm to be triggered, for real… for everything to happen… eh, make sure someone calls the emergency department and asks them to trigger the alarm… because then the emergency department will call the switchboard, the emergency department will call the stroke team, the emergency department will call radiology, just like they always do. Eh, so the important thing to know is that you should call the emergency department and ask them to do it, instead of calling the switchboard… eh, that’s crucial, because otherwise, you’ll get to radiology, and there’s no lab available, and there’s no stroke team there to give the – to give the treatment… eh, meanwhile… eh, the alarms that still go out on the pagers, when the switchboard just presses a button and it says “Rädda Hjärnan,” those pagers are also on the stroke unit, so there’s some… readiness for something to happen… but it doesn’t mean they go down to radiology just because the pager beeps. And what’s on the pager is only “Rädda Hjärnan,” there’s no information, you want this information about weight, personal number, and time… that’s why we have the phone call.

IB: Mm. I – I’m going to ask a question now that…

Inf2: Mm.

IB: …you’ve partly answered, but… I don’t want to interpret what you’re saying.

Inf2: No, no, go ahead – go ahead, yes.

IB: Ehm… [sighs] Are there departments in your hospital where you feel that the knowledge about this, both the knowledge and the experience, is particularly good, or where it’s lacking?

Inf2: Ah, absolutely. Most definitely.

IB: Can you elaborate with some examples?

Inf2: Particularly – particularly good is neurology and the stroke unit… mainly because there – the stroke unit especially. If there’s an alarm from there, you – you – they trigger the alarm themselves, and act as… as the ward staff essentially. Eh, you don’t need to involve the whole – you are the chain, so to speak. Eh, neurology… eh, the doctors working there are the alarm chain. The same – it’s the same… it’s a medical team, stroke and neurology, so even if the staff aren’t the same, the doctors are the same, so that solves the issue, they’re very good, yes! Eh, thoracic is quite good because they have many patients who wake up with strokes after thoracic surgery, they’ve gotten better and better at triggering alarms properly, aiming for thrombectomy.

IB: Mm.

Inf2: Eh… and the less acute somatic care you do, the worse you are… eh, so internal medicine, cardiology are quite good… ehm… eh, surgical, emergency surgical wards are okay. Geriatrics, psychiatry, quite bad. Sometimes, very bad. Eh, and – and also a… a mindset in… in like… Eh, my favorite, my personal favorite was, eh, I was called to geriatrics, because we were going to trigger a – and it became a Rädda Hjärnan alarm – and they were like: “We’re going to radiology now” [inhales through clenched teeth] “but we want to give all the others dinner first” [laughs] They – they didn’t get dinner… we actually went to radiology. But – but yeah, eh, yeah, the less acute somatic care you have… ehm, and the more toward internal medicine, so to speak, the better you are. The further from that, the worse you are… ehm, and of course, those of us who do this are good at it.

IB: But – but… a follow-up question to that then…

Inf2: Mm.

IB: Is… New colleagues, we’re not just talking about doctors but really all the healthcare staff, across the hospital.

Inf2: Mm.

IB: Is there any information provided, about the fact that there’s a routine and a plan for how things should be done?

Inf2: Yes, it depends… eh, or like this, the new nurses being hired left and right everywhere all the time, there’s no educational effort there at all. Ehm, all the new doctors – no, there’s no routine that all new doctors get this, but the largest intake is the junior doctors who then stay. They have an introduction week… and placements, where we try to ensure that everyone sees a Rädda Hjärnan alarm. Ehm, ehm… that doesn’t say much about the “in-house” routine specifically, no… but just to have the right mindset.

IB: Mm.

Inf2: Like, to – to think – the issue for us isn’t whether you trigger the alarm in one way or the other, it’s always more or less chaotic… and the fact that, because the decision… eh, you have to gather more information in a very pressured situation, in a way where you… and not see the patient… and you’re often new. That combination is unique. When it’s the ambulance, it’s the backup physician who makes the decision, and they’re used to it, so they don’t feel the pressure.

IB: Mm.

Inf2: In the emergency department, you see the patient in front of you, so it’s less pressured too… whereas this situation where you only hear about it… ehm, and often you’re younger too… ehm, it becomes a pressured situation, which, well… it takes a few extra minutes… as long as you realize it’s a stroke, and think stroke, and start in some way to get into the right… right mindset, so to speak… that’s where the time really is – like how much time passes from stroke onset until the first contact with someone knowledgeable about strokes… that’s where the big chunk of time is. Eh, and there it’s often not that you realized it now but thought correctly two hours later, but that you didn’t realize it until two hours later… like, it happened between checks, eh… that’s where the real time is lost, eh, and that’s something you can’t fix. Eh, but no, no relevant education package exists, no. Just that…

IB: But…

Inf2: From a cost-effectiveness standpoint… of everyone who comes in, very few will actually do this. And when they do, they don’t need to do anything special, they just need to know to talk to the right person. Eh, and that’s what we do with everything else too. I didn’t get an intro about who to call when someone has sepsis, I just learned that you call infectious diseases, from my colleagues. I mean – you – you learn healthcare, and this… well – if someone has a stroke on neurology, we call… and Rädda Hjärnan, we know about it. I mean, how much do we really need to educate… we – we could educate to shave off five or ten minutes on the “in-house” line, yes, we could… but, we’d have to educate so many people, so often, and repeat it so often for something that almost never happens… and the big time loss is usually that they’re found between checks, and then we’re talking hours, not five minutes. Eh, so like… no… we – we – we’re – we’re satisfied with it being half-good… at least I am.

IB: We’ve talked quite a bit now about [clears throat]… about what happens up until someone contacts… the right on-call doctor and I… I’m going to interpret what you’re saying: I get the impression that… you’re saying it’s difficult… to find these patients… and that it’s difficult to do anything to make it easier to find them. Have I understood you correctly?

Inf2: Yes… or difficult to find them… or like this, eh… or what do you mean by difficult to find them? For who? For us on stroke or for those who are responsible for them?

IB: I’m primarily talking about those responsible for the patients because you don’t go around checking all…

Inf2: No, absolutely not…

IB: … it’s the assistant nurses, physiotherapists, and nurses who see ward patients the most.

Inf2: Hm.

IB: It sounds to me like, despite them being there, there can still sometimes be delays of hours before the patient is found.

Inf2: Yes, or… yes – yes, it can, but that’s because they… they don’t check every patient every ten minutes. Eh, rather, many patients are checked very infrequently because their condition doesn’t require more… and yes, that’s exactly how healthcare works… eh, and just there, eh… it’s… if you put it like this: It’s common for an alarm – alarms start in two common ways… alarms, when they’re discovered to have this, and you know “last seen well,” because it was the last check or they’re witnessed having it, eh, because it happens in front of people, so to speak. Eh, what’s very uncommon, though, is that we get a… that we get a request for a stroke consult or something like that… eh, later, without the Rädda Hjärnan process having started… so the patients are definitely found, and at least in the past year – years, I don’t have great statistics on this, but yes – or actually, I just went through all the 2020 thrombolyses, we had about four “in-house” alarms, so to speak… so as soon as someone realized it was a stroke, or as soon as someone saw something… something reasonable, until we were on board, it was just a few minutes… like just realizing it quickly was a stroke… they triggered the alarm in such a way that we got going pretty quickly… eh, and then from… from the first realization to treatment is about forty-five to fifty minutes, something like that… eh, and – but just that you start the transport to radiology, you start several things, you have to discuss things… so even though we often do it in about twenty-five minutes when the radiology referral has already been written and you know what to do and just go straight in, eh… it takes a few extra minutes… so from first realization to actual thrombolysis, it’s not that bad. It looks catastrophically bad when you compare it to the twenty-five minutes from the emergency department, but that’s an unfair comparison.

IB: Who takes the patient to radiology?

Inf2: Eh, whoever, eh [interrupted] whoever is involved is smart enough to delegate [laughs]. Eh, in prac – so this, my advice to the primary on-call physicians is this… eh, you take two nurses, one nurse, eh, you tell them “you go with the bed now!” and preferably you start pushing the bed so it starts moving a little bit, really physically ensure that it starts moving… and then you delegate to another nurse: “Call the emergency department and ask them to trigger a Rädda Hjärnan alarm”… ehm, and those two then… so those two things happen in parallel, so you don’t have to wait for one or the other. But it’s the nurse, yes, the primary on-call physician… and usually, if the ward doctor is there, they tend to join in because they find it exciting…

IB: But they…

Inf2: Yes, also, yes…

IB: It’s the staff from the department that raised the alarm who…

Inf2: Yes, it definitely is!

IB: Right.

Inf2: They still own the patient until we take over… which we usually do if the stroke becomes a much bigger problem than what they had… or we thrombolyse or thrombectomize. But if it’s uncertain, it’s not even certain that we’ll take over the patient, just because the alarm has gone out doesn’t mean we’ll take over the patient.

IB: What happens then, like… after, we can take it from when… maybe the radiology images are taken?

Inf2: Mm…

IB: What happens after that?

Inf2: For these cases specifically or in general? Not that it’s any different…

IB: Let’s talk specifically about “in-house” cases.

Inf2: Well, what time of day is it? I mean, is it the middle of the night or not?

IB: You can talk about both scenarios.

Inf2: Alright, eh, yes, exactly… so during the day, when the “Rädda Hjärnan” alarm comes into radiology… and the referral has to go to radiology as well, it passes by the eyes of the radiology on-call physician somehow, so they’re on-site and do the “bleeding or no bleeding” interpretation as soon as the image is in the review station… eh, and is… eh, if the status and history are well-documented beforehand, a decision can be made on that information. The backup physician can then make a decision about thrombolysis or not, and then you just tell the nurse, eh, “mix it,” and if the reports are well-done, they’ll have an estimated weight, and they’ll mix based on the estimated weight… and in the meantime, we do a CT-angiogram, for thrombectomy, and also carotid stenosis for that matter… eh, and then we move to a room that’s right next to the radiology room and administer the thrombolysis there… eh, that last little step has shown to increase our treatment time by a minute or so, so we’re seeing if we should do it differently, eh, in the future… eh, but that’s how it works. If it’s the middle of the night, then there are two options: either the primary on-call physician is very skilled or has the backup physician there and feels they can make the “no-bleeding” interpretation themselves and act on it… eh, and then they do, then it goes just as fast. Or you wait for a call from the radiology on-call, which usually takes about four or five minutes… eh, when I’ve reviewed cases, like… what they write, at least, is four or five minutes… eh, and – and… it’s not great, but it’s not catastrophic either, by any means… eh, yes.

IB: How does it work if thrombectomy becomes relevant after that?

Inf2: Yes, exactly – then the CT-angiogram is done, and… and, yes, it depends on who you are, if you’re more on the primary on-call side and don’t have the backup physician there… if there are two of you, you should split up, with the primary on-call physician more going toward the patient, being with the nurse during administration, and completing the NIHSS assessment, which you ideally did somewhat conservatively to make a decision beforehand without… and ideally, you complete it afterward, inform everyone… meanwhile, the backup physician stays with the CT-angiogram image and starts making a preliminary assessment, perhaps themselves, perhaps not, depending on who they are, but otherwise involves neuroradiology and the interventionalist to interpret the CT-angiogram, ehm… so, yes… just that this happens simultaneously as the bolus is being administered, so you’re nearing the ability to make a decision… eh, if you know you’re in an extended time window, then you go straight to CT-perfusion, and then thrombolysis isn’t relevant either… so that solves that issue, in that way.

IB: Mm. [pause] I’m thinking… maybe again, so I don’t read too much into it...

Inf2: No, no, go ahead!

IB: But when you think about the acute care chain, the one you have for this patient group with “in-house” stroke, how do you feel overall that it works?

Inf2: As good as the difficulty and… as good as the mismatch between difficulty and… and skill… allows.

IB: Yeah… and if you…

Inf2: I mean… just that – and the mismatch is that the person who has the patient isn’t used to sick patients… we can’t fix that… so how good can it get when we have to work with someone who… isn’t very good at this… it can’t be much better than expected – it can never be great… it can be as good as expected, and I think it works about as well as expected, actually. If it were worse, then we’d have a lot more people getting CT scans first, going back, and then maybe calling later… eh, more would have delays and issues, and we’d never get going… eh, more would ask the next day: “They had a stroke yesterday, should we do something today?” I mean, the things we don’t see much of… those are the indicators that what we’re doing actually works. But as I said… the chaos that’s always there, I don’t think we can fix… unless someone has a brilliant idea we haven’t thought of yet, then – then I’d love to hear it… eh, but I don’t think we can get rid of the chaos, because of the mismatch between who has the patient and the difficulty of the condition.

IB: I still want to ask, though, if…

Inf2: Yes…

IB: [clears throat] if you had optimal conditions… or, how should I put it…

Inf2: Mm.

IB: If you had the ability to make changes… is there anything you see that you’d still want to do to improve things, in an ideal world, or however you want to phrase it?

Inf2: Yes, yes, exactly… in an ideal world, this would be… something you learn [interrupted] just as well as you learn our most critical procedures… I mean – yes… status epilepticus, I can do that by heart because I’m a neurologist, so that’s maybe… just that. But I can also do cardiac arrest by heart, and I can do anaphylaxis by heart… I’ve never seen anaphylaxis, but I know it by heart nonetheless. Eh, should you be able to know how to initiate a “Rädda Hjärnan” alarm by heart… yes, I’d love that. That would be nice. Eh, and… for both doctors and nurses, because it’s often the case that, especially in the evenings, the doctor isn’t on the wards to be called in to assess something, but it’s the nurse who’s there with the patient… eh, even if they involve their on-call line, that person is often elsewhere, so to speak… eh, so the nurses also need to know what to do here. That’s – that’s what I’d like! Eh, but… just how often we’d have to drill this, ehm… for the few who will actually do it, with only a marginal improvement, eh, is the reason we don’t do it. Beyond just having general information available, now and then, especially to those who ask for it.

IB: Mm.

Inf2: And maybe I’m just jaded and don’t care, I don’t know…

IB: I can still ask, to…

Inf2: Mm…

IB: To cover all perspectives, but have you received any improvement suggestions, or when such events have happened in the hospital, how has the staff involved expressed themselves about the course of events, and has anyone besides yourself suggested how things could be improved?

Inf2: Yes, no, no, absolutely, we… we’ve – we’ve, or like this… we’ve had this discussion, or nothing significant has changed in humanity… in the past thousands of years… eh, we’ve had this discussion many times, eh, and it always ends up in about the same place… eh, in the end… eh, so… however, what we’ve done recently is a bit better – we’ve printed an information poster, so to speak… for suspected stroke, the timings, like four and a half hours and so on, call this, otherwise call this… ehm… ehm, like, how to act – having that available to hand out during information sessions and other things, and just this, people are interested in stroke to the extent that… they come by for internal trainings and so on, it happens now and then, especially for those who’ve had more than one alarm every five years, so just this, we’ve had two cases now… now they want to see if they can improve, well then I’m there and talk… eh, but… eh, and the chance of having a quick discussion increases, but just this… the places that produce many cases, like those with the most severely ill patients, that’s really thoracic, which produces the most… they’ve already gotten pretty good at this. And the issue there is really that… eh, when – because it’s a sedated patient who’s waking up… when did this really happen? Is a… is, a difficult question… and how sick is the patient because you don’t know how bad they are after their heart surgery until you’ve evaluated that after a few days, so like… it, there’s a lot more hassle because of the patient group rather than because they’re bad at triggering alarms, they’re good at triggering alarms…

IB: So you’re saying…

Inf2: So just that… Yes, it was – or actually we had… there was just one, one brilliant idea that came up, actually… when we had this kind of… this kind of discussion with cardiology, and that was when we were trying to draw up a flowchart of how this works… and it was just, eh… no – cardiology is here, you have the patient, eh… you – you call the neurology on-call physician… that’s it! Eh, it was – it just kind of resolved itself in the room, on the spot, like, it shouldn’t be harder than this… just do this… just do this, and like, it’s so simple that… you don’t need to study, you don’t need to know, just know that you should just call, and it will sort itself out… eh, that’s – that’s what we’re broadcasting, which we try to do, as much as possible… eh, that… that’s the best way we have… simply… ehm, and then when you start talking, you work things out: “Will you write the referral,” “will I write the referral,” “should we start moving,” “should I see the patient first,” all that… depending on who the primary on-call physician is, what the facts on the table are… ehm, yes, the better the primary on-call physician, the clearer the stroke, the more you can do over the phone.

IB: Mm… [pause] Yes… Is there anything else you’d like to add?

[Pause]

Inf2: Eh, no, just that, eh… if I had a stroke myself, I’d want it to be an “in-house” stroke… because… because it’s my experience that “onset-to-needle” is better… or like this, if I want my stroke to be found, then I want it to be found on a ward, because the distance to radiology is shorter, eh… if I want to have a stroke in front of someone so that the alarm goes off immediately, I want to have it at the hospital [laughs], because – because things move faster… eh, no… so if I were to have a stroke… despite all the mess and hassle, in the end, they get their treatments early… ehm, sometimes remarkably early, the only ones I’ve seen get treated earlier are those who practically have their stroke in the car outside the hospital and just drive straight into the emergency department, that’s happened! Eh, but like, that’s the – because in that case, we have the right mindset, the right staff, and we just run in, so to speak… that’s the only time I’ve seen a better chance of a faster “onset-to-needle,” and that’s really what matters, not “door-to-needle,” it’s just a practical measure for us to be able to compete, which we should, because it makes things better for patients in the long run… but what really matters is “onset-to-needle”… eh, and the biggest part of that is beyond our control in healthcare, it’s controlled by the patient…

IB: Yes, well, then I think I’ll have to thank you for your participation!

Inf2: Absolutely!
