# Supplementary material for: Exploring strategies for management of in-hospital stroke in Sweden: A qualitative study
Source: PLoS One. 2024 Nov 26;19(11):e0313765. doi: 10.1371/journal.pone.0313765 (PMC11594569; doi:10.1371/journal.pone.0313765)
Supplement: S3 Text — (DOCX) [file pone.0313765.s004.docx]

**IB:** My first question is just to hear a little bit about who you are, what kind of residency or specialist training you have, and how long you've been responsible for... the stroke chain.
**Inf3:** I've been a specialist for almost a year now... in November it will be a year, in internal medicine, that is.
**IB:** Mm.
**Inf3:** I studied in [university town]… and then I did my internship in [city], then I worked for a year at the ENT clinic in [city] before moving to [university town] and did two years of my residency there in internal medicine… and then until I moved, stroke care in [university town] was shared between neurology and internal medicine… and just when I left, neurology completely took over the responsibility. Ehm, then I moved to [current city] and here we have everything. Ehm, and here, a little bit... since I had some experience in stroke, and mostly because there was a big gap here, there was one senior physician who now only does research, who was responsible for stroke care, and he hadn't cared much about the clinical side for several years [laughs].
**IB:** Mm.
**Inf3:** So there wasn't much... there were very old guidelines and no clear routines, etc. Ehm, yeah, so I started getting placed there, at least a little, and I found it interesting, so you get sucked into it. And then I became, I have been relatively frequently placed in the stroke unit during my residency. In the stroke unit here... I mean, we have three – as it is now, we have three medical wards and one geriatric ward… eh, and one of the medical wards is a half-dedicated stroke unit, so to speak. Then things happen... there’s never just stroke patients there… but usually, most of the stroke patients are there… and we also get neurologically focused patients there, so to speak.
**IB:** Mm.
**Inf3:** Post-neurosurgery, seizures, and… yes, things like that… and as I worked longer and he moved even more into only research, I became the oldest resident interested in stroke. There's another one after me… eh, who’s only a year behind me or so, but he's also interested in lungs, so we each have roughly two areas we focus on, trying to make things work. So, it was under the previous head, who was an endocrinologist [laughs]…
**IB:** Mm.
**Inf3:** So, we updated a lot of guidelines, including the stroke – thrombolysis – thrombolysis guidelines, ehm, which turned out really well, we reduced our door-to-needle time by half an hour in just a few months, and that wasn't because we were fantastic, it was because it had been really neglected.
**IB:** Mm.
**Inf3:** So, ehmm... I think it was mostly because we chose to reduce waiting time in the emergency department where people started taking blood tests and waiting… and then the head changed about half a year – a year ago… feels like everything has been a blur with COVID, but… and now it's [name] that I’ve written to you about…
**IB:** Right.
**Inf3:** He’s a hematologist… also very research-focused – he now does more than fifty percent research, I think… ehm, I think he's an associate professor or whatever he is... yeah… ehm, so he's the head, but he's not very… and he's the kind of person who, when it becomes necessary, thinks things through, looking up evidence and such, but it’s more when he’s forced into it… eh, so we haven't made many changes recently, and… and regarding this patient category, as you mentioned, in-house… we don't have… we don't have a guideline, and I wouldn’t say that we have... I don’t think we’ve ever had a discussion or a meeting in the clinic about how we handle these patients.
**IB:** Okay.
**Inf3:** Ehm…
**IB:** That would have been my next question, if there was a…
**Inf3:** Yeah…
**IB:** But if there's no routine, how are these patients handled in practice?
**Inf3:** Yeah, in practice… I thought you said those who have a stroke while hospitalized for another reason, are you also including those who are already admitted for a stroke and have a recurrence? Does that count as a deterioration during stroke monitoring, etc… or is it not that clear-cut?
**IB:** For the purposes of this study, where it’s about a routine, we can say that “yes, it does.”
**Inf3:** Mm.
**IB:** But if we look at how the definition will look later in a quantitative study, then no, it doesn't, because it's not registered in Riksstroke [national stroke registry], a recurrent stroke within 28 days is not registered. But I mean, a patient who’s admitted for a stroke and has a recurrence, they should undergo a CT and maybe consider some form of…
**Inf3 (interrupts):** Yeah, yeah, and be evaluated…
**IB:** …acute treatment, so yes, they count in this context.
**Inf3:** Mm… well, in practice… stop me if I ramble on and talk too much [laughs]…
**IB:** Absolutely!
**Inf3:** Because you’ll notice, it will probably come through all the time, as it already has, things I have strong feelings about, etc., and you can – I won’t take it badly if you say: “Okay, wait a minute, now you need to tell me something relevant for me.” I won’t take offense.
**IB:** Absolutely.
**Inf3:** So, well… if we think of those admitted to… let me start with the group admitted for stroke monitoring, which in practice, they’re monitored for 24 hours, and it’s been a bit of a struggle to get everyone to accept that they need to be monitored for at least 24 hours. Otherwise, for example, TIA patients who were completely fine could often… some would discharge them the next day because they were like, “They’re symptom-free, they’re fine now”… eh, but now all are monitored for 24 hours, and it’s every three hours, not spaced out… eh, and there it gets detected quite quickly. And I’d say that during the day, they will contact the responsible specialist on the ward first if something happens… or the resident, depending on the level of trust... how experienced the resident is if they’re placed there… if it's an intern [AT], they’d probably go straight to the senior physician…
**IB:** Mm.
**Inf3:** Ehm… and then… the rest of the wards, meaning the medical wards, they will probably also initially contact their senior physician, or if they’re unavailable, the day on-call senior physician… eh, and the rest of the hospital will contact the medical backup on-call physician… eh, so in internal medicine, it would be the responsible senior physician on the ward or an experienced resident if they’re placed there, and for the rest of the hospital, it would be the medical backup. Eh, and then…
**IB:** What happens then?
**Inf3:** Well then… eh, there is a "Rädda Hjärnan" (Save the Brain) alarm that’s triggered in the emergency department, but it's not established that it’s triggered from the wards… or from the other wards.
**IB:** Mm.
**Inf3:** So it will be – but it would probably be up to the individual backup on-call physicians how they handle it. I mean, they could call down… if they – I think the smartest thing would be to call the emergency department and say: "Can you trigger a Rädda Hjärnan alarm for this personal number?" That would be the most efficient thing to do. Eh, because then the chain of contact with radiology starts, eh and with the ICU we have… eh so that the ICU is ready to mix… I mean, they’re ready to mix, they don’t mix in advance so as not to waste it if it’s not needed, they’ve decided they can’t afford that… eh, and radiology is informed, etc., and everything is put on hold, so the CT is ready. But I guess that in many cases, the backup might choose to go and examine first and then call radiology and say: "Now we want an emergency scan" and then it becomes: "Oh, are we running this as a Rädda Hjärnan?"… "Yes, yes, we are," more or less… so…
**IB:** Mm.
**Inf3:** Eh, with a high risk that it becomes a bit slower, of course… so that’s probably how it would go.
**IB:** Mm. And, like… how… if I… I don’t want to interpret too much of what you…
**Inf3:** No [laughs briefly].
**IB:** Put value into what you’re saying… How do you think it works? Is this a good way, or a bad way? Or…
**Inf3:** No… no, it’s not a good way. It’s – no, I mean, already when you asked me, I was like: "Oh right, this is another area I should tackle." Eh, and I feel – yes, because I know even though I’m not technically responsible, I know that if I don’t push something, it won’t get pushed. Eh, so I don’t think it works optimally. I feel like I should… ehm, I should talk about – I should figure out myself what is optimal, what’s the optimal way to handle this – I tried to think that I wouldn’t rush ahead and make a bunch of changes and tell you: "Yes, now I’ve actually started doing this and that." I also haven’t had time for that, but… I think I should figure out what’s optimal and then ask… prepare the emergency department for the idea that you should be able to call and trigger this alarm from within the hospital too.
**IB:** Mm.
**Inf3:** Inform radiology and then take one of our Tuesday meetings, where we also have some opportunity for education within the clinic, and say: "Now I’m thinking we should do this." Get their input, because as the backup physician, I’m very new in that role, so it could easily come up that there’s stuff I know from experience that I haven’t thought about. Like, “Oh, what about this and that” and “how will surgery know,” and I’d also need to inform the other departments in some way too.
**IB:** Hmm. But – but if we go back a bit to the chain, because you say that it sounds like a stroke ward or a medical ward would probably first contact their, like... ward physician…
**Inf3:** Yes.
**IB:** and many other departments would contact the medical backup physician…
**Inf3:** Yes.
**IB:** and then you say that the medical backup physicians, or like… it might vary at that stage how you handle the actual alarm.
**Inf3:** Yes, exactly. Definitely.
**IB:** But let’s say after that – then they should go to radiology. Who takes the patient to radiology?
**Inf3:** Well. It will… if it’s on our side, it will be the same physician who is recruited, so to speak, the senior physician or resident, maybe an intern, and staff from the ward who will have to go. So that staff will need to be freed up, so to speak, saying: "Now we have to go." The – I don’t think that will be a problem, they’re used to… I mean, it will be handled like a deterioration of a patient, so to speak.
**IB:** Mm.
**Inf3:** Eh, so it will be from the ward that it happens.
**IB:** Hmm.
**Inf3:** The problem might be at night, though, when we have severe staffing issues, ehm nurses… we reduce the number of beds. We’ve just changed it so that we open and close a ward on the weekends, meaning close it for the weekend and reopen it the next week, just to free up some more beds during the week…
**IB:** Hmm.
**Inf3:** So, it’s really thin at night… that could be a problem, getting them to radiology, but I would say there’s an… it’s not a problem in the internal medicine department to grasp the urgency of this, to understand the urgency of it. It happens that Rädda Hjärnan patients end up on other wards too, because we’re so full sometimes… [background noise]. Do you hear the noise a bit?
**IB:** It’s okay.
**Inf3:** It’s fine… okay yeah…
**IB:** And how does it work from other wards then?
**Inf3:** From other wards then… definitely, they would contact the medical backup physician… eh, and I don’t think they would choose, I mean, as it is now, I don’t think… I don’t think many physicians on other wards would choose themselves to interpret that: "Damn, this is a Rädda… this must be triggered as a Rädda Hjärnan"... instead, they’d probably call the medical backup first.
**IB:** Mm.
**Inf3:** And – and kind of get confirmation – get it confirmed that: "This sounds like something we should trigger a Rädda Hjärnan for."
**IB:** Mm.
**Inf3:** That’s my guess, but it might be a bias on my part, but I also think that even if they wanted to trigger Rädda Hjärnan, they wouldn’t know… they would still need to think like I do now: "Okay, should we call the emergency department and say that?" So, I’m pretty sure they’d call the medical backup.
**IB:** Mm.
**Inf3:** Ehm, and if it was… if I was the medical backup and it sounded clear-cut from the description… it’s also about knowing the staff, but here you know almost everyone who works at the hospital…
**IB:** Mm.
**Inf3:** So, I’d want to say: "Go to radiology, can you take the patient to radiology, I’ll meet you there and examine them."
**IB:** Mm.
**Inf3:** Because that’s what I’ve tried to push, and what I try to emphasize whenever I lecture the interns about this, that the most important thing is you… you should only be looking for positive findings that are enough to indicate thrombolysis in the emergency department, that’s the only thing that should happen in the ambulance bay, as soon as you have enough with a lasting handicap, you should move on to radiology… you shouldn’t be placing IVs, taking blood tests, not… unless there are obvious contraindications, move on to radiology, and while in radiology, continue the examination and go through the contraindications while the CT is being done, we have nothing to lose by doing a CT, the chain must always keep moving forward.
**IB:** Mm.
**Inf3:** Until there’s a clear reason to stop… that’s what I’d do, but I think many others would choose to go there and examine first and then make decisions from there… and then… very different, very different for different backup physicians in terms of what they’d do next.
**IB:** Mm… You talk about this in a way, ehm [clears throat]… like in a way you think would happen.
**Inf3:** Yes.
**IB:** Can you recall a specific case, like what actually happened?
**Inf3:** Eh, no, I mean… no, honestly, no. I mean… on the ward that I’ve heard of afterward, or on the ward I’ve had, I’ve probably called… been involved in, but I… I, it probably hasn’t happened to me yet as the backup physician that I’ve been contacted for this, on another ward. I’m only day backup so far. Eh, and… no, I… deterioration though, of patients, recurrence, that has happened, and I know, then I’ve called radiology… eh, but that’s not… but there hasn’t been a recurrence… I don’t think there’s been a recurrence within 4.5 hours… clearly, I don’t think so. It’s been more about thrombectomy… eh, as the plan in those cases.
**IB:** So you mean that they haven’t discovered it within?
**Inf3:** No, not like that, it hasn’t happened within, as far as I remember, it hasn’t been a clear recurrence that’s been within, like a patient who wasn’t thrombolyzed initially and then deteriorated within 4.5 hours from the original ictus.
**IB:** Right.
**Inf3:** I don’t think that’s happened. From what I remember. Ehm…
**IB:** Let me just ask about… identification, ehm…
**Inf3:** Mm.
**IB:** It presupposes that you, that you know what a stroke is and that there is acute treatment available and such.
**Inf3:** Mm.
**IB:** And I don’t doubt that the staff on a stroke ward is… like, drilled in this. But how about the rest of the hospital? Surgery, orthopedics, the operating room? Do you have any sense of…
**Inf3:** Well… I mean, I know, I know that our interns, overall I think, maintain a very good competence level, so as long as they’re on the ward, which they usually are, on those other wards, or yeah… I mean, on surgery, orthopedics, and medicine, eh… so I think there’s good competence in noticing those things. Ehm… then in the other areas, like in gynecology and pediatrics, well pediatrics probably wouldn’t have any, but in… gynecology and psych – yeah, psych I wouldn’t have much confidence in… I mean, they really shy away from anything somatic, having to take responsibility for any kind of somatic monitoring.
**IB:** Mm.
**Inf3:** So I wouldn’t have much confidence in them detecting things. Ehm…
**IB:** And among the rest of the staff then?
**Inf3:** [Pause for about 30 seconds] …there’s no activity, as far as I know, we haven’t been around to inform or held any “staff meetings” as I’m now thinking that this is something I should do.
**IB:** [First name], it froze for about half a minute, so I haven’t heard anything up until now, sorry.
**Inf3:** No problem, it…
**IB:** I asked about other healthcare staff,
**Inf3:** Yes.
**IB:** I don’t know if you…
**Inf3:** Yes, precisely, I answered… but it’s speculation for me too, it was psychiatry, maybe you heard that part, that I wouldn’t trust them, that they avoid taking responsibility for somatic issues.
**IB:** No.
**Inf3:** So I’m not sure they’d even detect pretty obvious things. Or rather, they might explain it away as something else. But otherwise, I have a general confidence in the competence. The interns are generally very competent, so they’d notice it. But as far as I know, we haven’t, during the time I’ve been here, informed the other clinics about this in any way… or made any kind of… I’m thinking now, based on my contact with you, that… it would be a good thing to bring up at, there’s something called a “staff meeting” here, which before COVID was held once a month where you could come and have lunch, the whole hospital, in a small conference room with chairs against the walls and… and each clinic would take turns presenting something. That would be perfect to do, like now we’re going to go over the FAST test or “I’m going to tell you what the current process is.”
**IB:** Mm.
**Inf3:** Because I definitely think there’s a lag in knowledge, if you think about knowledge of thrombectomy and what it offers in terms of possibilities, I think that’s very much lacking. For example, I know that there are even senior physicians in internal medicine who, if you asked them today, like: “How long… up to how many hours is there an indication,” I don’t think all of them would know about the 24-hour window, like, the possibility for selected patients.
**IB:** No.
**Inf3:** And then… yeah, the rest of the hospital is probably even further behind. And that’s because – I don’t want to give the wrong impression, but I think the hospital as a whole and my clinic, in general, with some exceptions, of course, people are… like, competent doctors, generally speaking. But it’s just so incredibly broad in internal medicine at a small hospital…
**IB:** Mm.
**Inf3:** That it’s impossible to stay up to date. Like, my hematological competence is really poor, yet I’ll have whole weeks with a ward where half the patients are hematology cases. So you just have to manage and figure out what you need to ask about, and so on.
**IB:** Mm.
**Inf3:** But when it comes to acute care, what’s new, what’s current, it’s just… it’s impossible to stay up to date, especially since we have such a high patient turnover. I mean… we never… none of us has any scheduled admin time, so we’re drowning in it. It feels like there’s too little time to educate oneself, let alone further educate the colleagues, and even less the rest of the hospital… So, yeah…
**IB:** I want to ask one more thing about the chain of care itself…
**Inf3:** Yes.
**IB:** Have you received – have you gotten any feedback from a colleague? Like, a doctor, nurse, or occupational therapist, physiotherapist?
**Inf3:** Mm.
**IB:** Like, someone who has been involved in such an incident. Have they had any suggestions for improvements or general comments?
**Inf3:** Well, I don’t remember clearly enough. I… I’d need to fill in the blanks. But I don’t remember… I haven’t received it while I’ve been seen as responsible, so to speak. I think I remember there’s been talk about it and feedback on patients who’ve had a stroke while admitted and someone pointed out: “That didn’t go completely smoothly,” like, “That could’ve been faster,” “That should’ve gone faster.” Not concrete improvement suggestions, and certainly not concrete plans on how those improvements should be implemented.
**IB:** No.
**Inf3:** No.
**IB:** But if I ask you instead then, if you were to speculate freely…
**Inf3:** Yes.
**IB:** I’ll ask it almost rhetorically and a little stupidly: Do you see any critical points in the chain of care where delays could occur, that might be overcome with a routine?
**Inf3:** Absolutely! [laughs]
**IB:** Let’s hear what you…
**Inf3:** Yes, well, first, making a decision and communicating the decision about who such an alarm should always go to. And then I think, in the absence of anyone else, it should be the day backup physician, in our case. Eh, because the only alternative would be to link it to the senior physician responsible for the stroke side, but I know that… eh, sometimes it happens that the senior physician has to take both sides of that ward, so they have 18-20 patients by themselves, and they can get completely caught up with other things, perhaps.
**IB:** Mm.
**Inf3:** Plus, whenever there’s sick leave, there’s a reshuffling, and then it could be that the contact number listed for the responsible senior physician might be wrong, like, it could be well into the day before you realize “Oh no, it’s not [name] today because of illness, it’s [another name].”
**IB:** Mm.
**Inf3:** So the only consistent… I mean, the only person-independent role is the day backup physician, and the switchboard always knows who that is.
**IB:** Mm.
**Inf3:** So I think that would be the best solution. Eh, and then, again, decide how they, in turn, should start the process, that it’s not up to someone to reinvent the wheel each time but that you take the decision to contact the emergency department internally, like, contact the emergency department and say: “Trigger a Rädda Hjärnan alarm for this personal number,” just like is done when an ambulance is on its way in for the same thing… so I think that would remove the uncertainty where staff doesn’t know who to contact from the ward, that they waste time contacting the wrong person, and then the next person doesn’t make an optimal, fast start of the process, so that… even if they manage to arrange a fast CT, the ICU might not have started preparing the medication, or there’s no staff ready to go down to radiology to give the thrombolysis. And so on…
**IB:** Mm.
**Inf3:** And likewise with the ambulance service for transport, because in the end, for these patients, you should be prepared to transfer them to [university hospital] for thrombectomy, if it’s within the window, if they find a large enough clot…
**IB:** Mm.
**Inf3:** So that would probably be good! And then, to actually make this work, I’d need to make sure there’s proper education, both within our clinic and in the emergency department, because there’s a high turnover there as well. Everyone who can take the role of the “lead nurse,” as they call it, who handles these alarms, needs to know this, otherwise, they’ll be wondering what this routine is.
**IB:** Mm. How could such education look?
**Inf3:** Well, in our clinic, there’s already an established… routine that we have internal education for all doctors, including interns, on Tuesdays. And someone is responsible for that and has an hour… no, you have an hour. So there would be an opportunity for me to reach out in a good way, and maybe I’d need to do it a few times to have a good impact, because there are many… at least one-third of the time, people don’t have time to attend because they’re too busy.
**IB:** Mm.
**Inf3:** So, I’d need to do it a few times. Maybe combine it with an email to everyone, and then… eh, and then, of course, put it in our own guideline folder, because we have a folder with guidelines that everyone knows about.
**IB:** Mm.
**Inf3:** Eh, and then, everyone in our clinic, I mean. And as for the emergency department, I know they also have regular training sessions, ehm so… but the last two years have been an exception, a negative exception… the plan was for me to be in the emergency department to keep the new thrombolysis guideline going. As I said, it was a great improvement at first, but then we’ve seen the door-to-needle time climb up again, and that’s because there are many patient categories, and there’s a high turnover of staff in the emergency department. So the idea was that it would be good if I could go there and do regular training… to keep it fresh. And that hasn’t happened during COVID because we’ve been told we can’t meet, and the emergency department has been overwhelmed just trying to staff the place due to staff being sick with COVID, and so on.
**IB:** Mm.
**Inf3:** So it’s been the worst possible circumstances over the past year, but…
**IB:** Right… Yeah, this has been really valuable for me to hear.
**Inf3:** Eh, yeah.
**IB:** I’ll try to summarize briefly. But we’ve talked a little bit about the fact that – that you don’t have a routine [clears throat].
**Inf3:** No.
**IB:** And it sounds like depending on which department the patient is found in, this is handled a bit differently.
**Inf3:** Yes.
**IB:** Eventually, the medical backup physician may still get a call, but that person has to come up with how to trigger the alarm, and it happens in different ways each time.
**Inf3:** Yes.
**IB:** And it sounds like you – or you say directly that: “This doesn’t work very well.”
**Inf3:** No.
**IB:** There are at least areas for improvement, you say.
**Inf3:** There are… yes. Then I should say that… the advantage, why… you might think, “This sounds like a mess,” but… Again, it’s because we handle so many different categories, and so many potential patients who deteriorate, too. I mean, we handle everything from this to oncology patients who get tumor lysis from their treatment while admitted, to heart attacks, of course, and to sepsis alarms and… it becomes such a mix… so to be the backup physician here, it requires that you just solve things, that’s the whole thing. It’s about being a constant problem-solver, so to speak.
**IB:** Mm.
**Inf3:** And coming up with solutions. We’re pretty good at coming up with quick fixes, and of course, we all know each other at the hospital. You know most of the phone numbers by heart, I recognize the voice on the other side as soon as they speak, so… In that way, the conditions for reinventing the wheel each time are relatively good, but of course, it’s a minus that there isn’t an established chain.
**IB:** Mm. I just thought of something now while I was summarizing a bit, just so I’m clear on this but…
**Inf3:** Yes.
**IB:** When everyone’s finally at radiology, and you’ve said that the ICU staff, they mix and then come down to…
**Inf3:** They prepare… they… they draw it up, or rather they prepare, like, set it up in front of them at the ICU.
**IB:** Mm.
**Inf3:** And then, when… when I’m down in radiology and eh… it hasn’t… if there’s enough with symptoms, with a lasting handicap, so to speak, and no contraindication has emerged, then as soon as I hear from radiology… I mean, you stand there looking at the screens with the radiologist and they say: “No, there’s no bleeding.” Then you call the ICU and tell them to mix. And then they mix the medication up there and then come down, it’s an elevator and a corridor in between. So then they come down, we stay in radiology because that’s what’s been confirmed, or shown to be the best practice, because we’re usually doing an angio while waiting for them to arrive. And… sure, it’s happened that we’ve passed each other in the elevator once or twice before it’s been fully confirmed, but we never leave radiology, even if the ICU is delayed, we wait for them. And they administer it during the angio, usually, and then they start the infusion…
**IB:** Right.
**Inf3:** But there’s a small… I mean, the mixing and coming down, it adds about 5 to 10 minutes extra, until the patient gets the treatment. And we’ve asked, like, how much is used, like, if we mix it when we first triage the patient in the ambulance bay. If they start mixing then, it would probably eliminate most cases where it turns out: “Oh no, this patient had some weakness before and just had an infection,” or it was a misjudgment or maybe it was a seizure. So you eliminate all those cases so that not too much is wasted. But… they haven’t changed it, because it’s 10,000 SEK per dose or something like that.
**IB:** What happens after it’s administered, thrombolysis, I mean?
**Inf3:** Eh yes, well… it’s a bolus first, and then an infusion over an hour. So, the bolus is given down there, and then you usually go up to the ICU. Because even if there’s still a thrombectomy question, the patient needs to be prepared for transport in those cases. Eh… a little bit… they insert an arterial line, or whatever it is.
**IB:** Mm.
**Inf3:** Eh, and we need to be in contact with [university hospital], so we need to call them if we see a large vessel occlusion, we call the stroke on-call there. We sometimes contact them even for regular thrombolysis, depending on who the backup physician is. But usually, we thrombolyze ourselves, unless it’s a very unusual case.
**IB:** Mm.
**Inf3:** But… then they talk, and then they have to talk to the interventionist on call and so on, that part can take a lot of time, lots of back and forth. We’ve had problems with… until three or four years ago, the coverage for thrombectomy was about one in four weeks.
**IB:** Mm.
**Inf3:** Then it was almost full-time for a couple of years, and now I understand it’s become less frequent again. So, there isn’t always someone available. So you need to first find out if there’s anyone who can do thrombectomy. But yes, otherwise, you go up to the ICU, and the patient stays there for the first… now I’m unsure… they stay in the ICU for the first 6 hours as routine, eh and then they go to the stroke unit. And then there are a few more frequent checks on a thrombolysis patient, more frequent blood pressure monitoring, etc. And it depends on how the patient is doing. If, for example, they need repeated doses of Trandate [blood pressure medication], then the ICU usually keeps them longer.
**IB:** Mm.
**Inf3:** But the basic idea is that when the patient is fully examined with the CT and angio, you go up during the thrombolysis.
**IB:** Would you say… I mean, the patient who has the stroke in the hospital…
**Inf3:** Mm.
**IB:** Does medicine or the stroke unit usually take over, or how? How does that work?
**Inf3:** Yes, yes, I’d say so. I mean, yes… on average. Of course, they could have some very severe other condition that keeps them on their current ward, but it’s hard to get the surgeons or anyone else to keep a patient who has recently had a stroke.
**IB:** Mm.
**Inf3:** So, yes, I’d say that we take over them.
**IB:** Ah, well, I feel like I’m pretty satisfied with my questions, I think I’ve gotten a good picture of how you… is there anything else you want to add?
**Inf3:** No, but it’s good that… I think it’s a good project you’re doing. It would be interesting to hear how others handle this, and for me, this has been a wake-up call to think about it a bit.
**IB:** I’m glad you think so. You’ll get feedback on how the study turns out!
**Inf3:** Feel free to get in touch if you think of anything else. Email or call is fine.
**IB:** Absolutely.
