# Supplementary material for: Exploring strategies for management of in-hospital stroke in Sweden: A qualitative study
Source: PLoS One. 2024 Nov 26;19(11):e0313765. doi: 10.1371/journal.pone.0313765 (PMC11594569; doi:10.1371/journal.pone.0313765)
Supplement: S4 Text — (DOCX) [file pone.0313765.s005.docx]

**IB:** Alright then. May I ask, [name], what is your specialty?
**Inf4:** I am an internist and neurologist.
**IB:** Yes, and you're responsible for "Rädda Hjärnan" [Save the Brain] at [hospital]?
**Inf4:** Yes.
**IB:** How long have you been in that role approximately?
**Inf4:** Twelve years.
**IB:** Yes, okay. My project is about patients who suffer a stroke while already admitted to the hospital for another reason. So, I wonder, at [hospital], do you have a routine for how to act if a patient is suspected of having a stroke? A patient who is already admitted.
**Inf4:** Yes, there is a specific routine for thrombolysis alarms for inpatients, where we have a chain of how to communicate with the stroke unit and how we initiate the thrombolysis alarm and proceed from there.
**IB:** Could you briefly describe what those steps look like?
**Inf4:** Eh, it's like this, yes, it’s any medical staff, whoever notices that the patient has symptoms, of course. And then they call the nurse who is the stroke coordinator, who takes a sort of questionnaire, you could say, with the caller, following stroke alarm protocols. And if the patient meets the criteria for thrombolysis, then we start a thrombolysis alarm. Then they call either the stroke on-call physician or, outside regular hours, the primary on-call doctor, who is a mid-level doctor working in the ER throughout the night. And then we continue with the thrombolysis chain. A special… if it’s approved, usually the doctor checks the medical record to see if there are any contraindications. If it’s approved, we take the patient to radiology, and they meet with the doctor who performs a status check, reviews the images with the radiologist, and then if thrombolysis is given, we move the patient to the [stroke ward] to administer the thrombolysis.
**IB:** Mm.
**Inf4:** The problem arises if the patient doesn’t meet thrombolysis criteria or is admitted for problems that might outweigh the stroke issue, which also happens. It could be postoperative patients, heart attack patients, and so on. In those cases, it’s more individual.
**IB:** Mm. So, if I understood correctly… the staff member who detects or suspects that the patient has had a stroke calls a nurse who is the stroke coordinator?
**Inf4:** Exactly.
**IB:** Is that 24/7 or…?
**Inf4:** Yes, 24/7.
**IB:** And then it’s that person who assesses whether they think it’s a stroke or not?
**Inf4:** It’s an assessment according to the stroke routines.
**IB:** Mm.
**Inf4:** And if the problem is deemed significant, then it goes on to the primary on-call doctor for a general medical evaluation.
**IB:** Yes.
**Inf4:** However, if it meets the criteria for thrombolysis, then we proceed in cooperation with the neurology department, as a stroke alarm.
**IB:** And then this nurse says: “This meets the criteria, so you trigger a stroke alarm”?
**Inf4:** Yes, yes. The difference is that we have a nurse who can write a referral to radiology and directly inform them that the patient is coming. So we bypass any delays caused by waiting for the doctor's assessment before we start radiology.
**IB:** I see. And if this is the routine, do you think that’s how it works in practice?
**Inf4:** I can’t say for sure because in practice, it’s very rare for patients to have a stroke while already admitted. We have almost 100% coverage on the neurology ward, meaning almost all stroke patients are already admitted to the stroke unit. And getting a stroke while admitted to other units, unless it’s vascular surgery, is very uncommon.
**IB:** Mm.
**Inf4:** So, we have routines, but it’s maybe 1-2 cases a year that we have to deal with.
**IB:** Mm. [long silence] But I’m thinking, since you’ve been here for many years, do you feel that the staff on other wards in the hospital are aware that this routine exists? Is there an awareness in the hospital that this is how to act?
**Inf4:** Yes, absolutely. Absolutely. Even if they call the wrong number, they’re directed by the primary on-call doctor because if someone isn’t fully aware, which can happen, they call the primary on-call doctor, and the primary on-call doctors aren’t often trained in stroke alarms, so they redirect immediately.
**IB:** Mm. And may I ask, even though there are few cases, do you feel… have you received any feedback from colleagues when this has happened, or from the staff on the stroke ward… do you feel the chain of care works well? Is it fast and efficient compared to patients who come in the usual way via ambulance through the ER?
**Inf4:** Yes, not worse, at least. I think the awareness of the routine is quite good and…
**IB:** Mm.
**Inf4:** It works. At least the first steps work very well. Then there can be some issues, because with more complex care, we have to ask [university hospital] for help, and then it’s a matter of communication, image transfer, and also transportation.
**IB:** Mm.
**Inf4:** So yes, it can become more difficult the longer it goes. But the first 10-15 minutes, up to maybe 20 minutes, if the resources are there, it works well.
**IB:** May I ask then, when this nurse assesses that it meets the criteria and the alarm is triggered…
**Inf4:** Mm.
**IB:** Who takes the patient to radiology?
**Inf4:** It’s the staff on-site who are responsible for taking the patient to radiology, where they meet with a team from the neurology department.
**IB:** And no one assesses the patient before they are transported to radiology?
**Inf4:** Not more than the preliminary assessment… wait a moment [goes to open the door to the office].
**IB:** Yes… [pause for a minute, Inf4 returns].
**IB:** Okay, I understand. And once the patient gets to radiology… or rather, does radiology also get the alarm so that they are aware…?
**Inf4:** It’s like this. In principle, we prioritize performing the CT scan before the doctor’s assessment.
**IB:** Mm.
**Inf4:** The idea is that a CT scan, especially without contrast, can’t harm the patient. However, delays due to transport, communication, and discussions are completely unnecessary. The patient is met by everyone involved in radiology, first we do a CT scan, and as soon as it’s done, without waiting for the radiologist’s interpretation, we proceed with the neurological exam and perhaps a review of the patient’s history, and then we make a decision based on the radiologist’s findings. And it works well.
**IB:** Where do you administer the thrombolysis, if needed?
**Inf4:** Unfortunately, we have to move the patient to the ward, which takes 5-10 minutes. It’s maybe 100-200 meters and two floors by elevator.
**IB:** Mm.
**Inf4:** And we have… when we are done with the decision, if the patient is to receive thrombolysis or is eligible for thrombectomy, if thrombolysis is appropriate, we prepare the treatment. You have to dilute Actilyse…
**IB:** Mm.
**Inf4:** And the patient arrives on-site, and then we administer it. Here we have some issues.
**IB:** It sounds like there might be a bit of a delay there, after the decision itself.
**Inf4:** Yes, but in practice, we lose the most time between the completion of the exam and the start of treatment. The first steps, the alarm, the information, the action, that all goes very well. Even the status check. Then different situations arise.
**IB:** May I ask, I’m thinking… now this happens rarely, but… before the patient is discovered, like… have you experienced cases where it’s suspected “maybe this could be a stroke?” or something else but the staff might hesitate on the ward, maybe unsure, and wait a few hours to see if it gets better? You know what I mean, have you experienced that happening?
**Inf4:** Unfortunately, it’s more the opposite.
**IB:** Okay?
**Inf4:** They call too early, more often than not, because everyone is… everyone is, how should I put it, aware that there is a thrombolysis alarm, thrombolysis treatment, and that time matters. So people tend to call too early rather than too late.
**IB:** Mm.
**Inf4:** Another thing is there are some strokes discovered by chance, for example, a patient wakes up after anesthesia.
**IB:** Mm.
**Inf4:** After undergoing a procedure. Yes, then it’s a different situation. Or the patient may be cognitively impaired and slept through the night, and then night staff come in for hygiene or another visit. The patient isn’t monitored, and it turns out by chance that something happened during the night, like a wake-up stroke.
**IB:** Mm.
**Inf4:** We don’t have the ability to constantly monitor all patients who might have a stroke in all 250 beds.
**IB:** No, no.
**Inf4:** But I think, as soon as trained staff come into contact with the patient, then there isn’t much delay. There’s no situation where they wait or refer to someone else, or wait for a doctor, no, no. There’s immediate feedback as soon as they’re done with their observation.
**IB:** It sounds to me overall like you think the chain of care works pretty well?
**Inf4:** It works pretty well! I’m convinced of that. It’s also reflected in the statistics… You know I also work as the senior on-call doctor, so I know how things are handled.
**IB:** Mm.
**Inf4:** So I think, no… it’s very rare that we have any [internal reports] due to delays or hesitations to call someone competent, so… that doesn’t happen.
**IB:** I understand. And… you say that at radiology, when the images have been taken, a small team gathers? Do you feel that the people there have the right competence to make decisions, like a specialist in internal medicine or neurology who can decide on thrombolysis?
**Inf4:** Yes, that’s a problem because… I think there are mostly… there are two main groups that participate in the thrombolysis alarm here. One is the stroke on-call physician, who is usually a specialist in neurology or a specialist in geriatrics, but they have handled strokes for many years together with us, or a resident in neurology. That group has very good knowledge of the routines and is really the quickest and most confident. But outside of office hours, it’s the primary on-call doctor’s responsibility, so when we had junior doctors as primary on-call, it was very difficult and complicated, they were unsure and unconfident in their assessments, with lots of back-and-forth and delays.
**IB:** Mm.
**Inf4:** Now it’s at the level of a resident or specialist… and since they’ve been working for several years, there isn’t much they want to discuss. The problem arises when something unexpected happens. For example, you have a brain CT that doesn’t show bleeding or stroke, but shows, for instance, a suspected aneurysm. Then you start wondering how to interpret it.
**IB:** Mm.
**Inf4:** Or other anamnesis information that unfortunately falls outside the well-structured chain. So, as soon as we call for upper-level consultation, at [university hospital], things get more complicated. They want this and that and think this way and that. The shorter the decision-making process, the less we seek help from others, the more we rely on ourselves, which is the purpose of thrombolysis.
**IB:** Mm.
**Inf4:** Thrombolysis isn’t a task for specialists, it’s a task that lies within the doctor’s competence, but not the highest level. So… as long as we manage on our own, it goes very fast and smoothly.
**IB:** Another question. Have you heard from staff, anyone who’s been involved in such an event, and given feedback, suggestions on how you should handle things differently? Have you received any such input?
**Inf4:** Absolutely! For example, my colleague called me because the patient had an aneurysm on the CT, and in our routines in our county, there’s nothing about aneurysms. But if you read up on it, it’s a relative contraindication.
**IB:** Mm?
**Inf4:** So he wanted to confirm his… so there was a lot of discussion because it wasn’t enough to call [university hospital], even the doctor there wanted to look at the images and check with their senior on-call, and get back, talk to the neurosurgeon and so on, it was a disaster. And then you read the literature that sometimes you give thrombolysis with an aneurysm, even if you don’t know it’s there because not all are detectable on a standard CT. What do you do then? You give thrombolysis and nothing happens. And then there are all these borderline cases, with borderline blood pressure, uncertain data about NOAC [novel oral anticoagulants] treatment, or other treatments like Brilique, and they feel unsure… or someone finds a meningioma, and someone wants to be more thorough than necessary and tries to get a second opinion on how to handle this with thrombolysis, and so on. Or with dissection, instead of giving thrombolysis, they want to call a higher authority and check…
**IB:** Mm, mm. Naturally, there can be difficult cases in terms of whether to give treatment or not.
**Inf4:** One out of three patients is a bit more complicated than the others, and if you’re inexperienced or unsure, it can delay things a lot.
**IB:** But there’s been no feedback about the chain of care itself? Like, “we think you should do it this way instead,” or…?
**Inf4:** Eh. You know, we started thrombolysis in 2012, so you might not have been in practice for long. Ah, I have to say, at that time, there were very strict rules for intravenous thrombolysis and thrombectomy was very rare.
**IB:** Hm.
**Inf4:** It was fairly simple for 6-8 years, then somewhere before the pandemic, we started seeing more wake-up strokes, the extended time window for thrombectomy, and so on. Then it started getting a bit more complicated. At the same time, the increased use of various anticoagulants made it even more complicated because there were questions about the exact number of hours since the last dose, “16 hours, 20 hours, maybe they didn’t take NOAC yesterday, what should I do,” and so on.
**IB:** Hm.
**Inf4:** You can always ask new questions. Then came the now-active thrombectomy-thrombolysis chain with several possible outcomes and now multiple thrombolysis protocols, you won’t find a single place where the thrombolysis routine fits on one page.
**IB:** No…
**Inf4:** Four to six pages. People want to describe all possible outcomes, all possible preemptive reactions on paper. This has led to it no longer being a protocol, but a bible. If you’re the primary on-call doctor at night, you’re working a twelve-hour shift, you’re alone at the hospital and you have ten patients in the ER, how much focus can you give to the thrombolysis patient?
**IB:** Mm?
**Inf4:** And you get a thrombolysis patient maybe once a month, or once every two months because you’re not a neurologist. We can’t staff a neurologist on duty 24 hours a day, every day of the year, it’s just not possible.
**IB:** No… no.
**Inf4:** So yes, I think forward… we need to simplify the routines that exist to make it easier for the doctors working on the front line to make decisions.
**IB:** Mm, I understand. But just to summarize briefly. I understand that there is good awareness of this routine at your hospital. The staff knows they should call a specific number, and they reach a stroke coordinator, a nurse who triages the patients based on established routines, and if that person sees that the criteria are met, a stroke alarm is triggered in the hospital, where things proceed in a standardized way. The staff takes the patient to radiology, a team arrives at radiology to assess the patient, and sometimes before the assessment, the patient has already undergone a brain CT.
**Inf4:** Yes, as a rule, the CT is done first, and then an assessment is made regarding thrombolysis.
**IB:** And then when the decision is made to administer treatment, you need to transport the patient to another ward…
**Inf4:** From radiology, yes.
**IB:** Do you usually perform angiography sequences before transporting the patient?
**Inf4:** We do what's called a single-phase angiography, a neck angio, because it’s fast and simple and provides the most information. And that leads to some problems because, ah… first, the patient may arrive without weight data, and you need to know the weight. Ah, then it’s a phone call. The next thing, the patient is moved out of the CT scanner and examined, and then the decision is made to proceed with the alarm, which means automatically proceeding with the CT neck angio. And then you have to place the patient back very carefully in the scanner, and I’ve noticed that it takes a lot of time for radiology to set all the markers, stabilize the patient correctly.
**IB:** Mm.
**Inf4:** Then you start the contrast. And if you succeed and have a good IV line, that’s great. But sometimes a better line needs to be placed. Sometimes the patient can’t tolerate the speed and vomits, and so on. It’s a bit troublesome. On the other hand, it would be tempting to start thrombolysis and, with ongoing thrombolysis, start the CT neck angio, but nowadays, for psychological reasons, it’s not possible. Do you know why?
**IB:** No.
**Inf4:** No. We had a case with new stroke symptoms, and they were about to administer thrombolysis, all the criteria were met. But on the CT neck angio, it was revealed that the patient had a newly discovered, completely asymptomatic aortic aneurysm with dissection that extended into the neck vessels. If they had received thrombolysis, it would have ended very badly. After that case, we decided always to perform the CT neck angio or some other form of contrast imaging and wait for the results before proceeding with thrombolysis. It’s not based on statistics, it’s psychology.
**IB:** May I ask, just at your hospital, what’s the reason that you don’t administer thrombolysis in radiology?
**Inf4:** You would have to bring the medication there, prepare it on-site at radiology, and you wouldn’t be able to start it there. Then at radiology, you would also need to monitor the patient during thrombolysis, you would need to place a urinary catheter before thrombolysis, and there’s no space or resources at radiology. It becomes more complicated. In summary, if you calculate what’s fastest, it’s to take the patient to the [stroke ward] and start there, which takes 5-10 minutes if everything goes well.
**IB:** Mm.
**Inf4:** Instead of bringing everything there, setting it up, and then going to the ward with ongoing thrombolysis.
**IB:** I see. I don’t think I have any more questions. It was nice talking to you, and I thank you very much for your time. Would it be alright if I contacted you again if I have any more questions?
**Inf4:** Absolutely! Also, I’m very curious about what you’ll find in your research, so when you’re done, feel free to send me your conclusions.
