# Supplementary material for: Exploring strategies for management of in-hospital stroke in Sweden: A qualitative study
Source: PLoS One. 2024 Nov 26;19(11):e0313765. doi: 10.1371/journal.pone.0313765 (PMC11594569; doi:10.1371/journal.pone.0313765)
Supplement: S5 Text — (DOCX) [file pone.0313765.s006.docx]

**IB**: …and through Riksstroke, I've received information that you are the stroke responsible doctor, is that correct?
**Inf5**: Yes, that's correct!
**IB**: But… can you tell me, do you have a routine at [hospital] for what to do if a patient has a stroke while already admitted to the hospital?
**Inf5**: Mm. We have a routine right now, eh… it will be revised within the next few months. We are currently doing a major review of the entire stroke process and… we have started at the other end with the large group, that is, those who come in acutely.
**IB**: Mm.
**Inf5**: But the changes we are making there will also affect “in-house” thrombolysis, so we will need to go through that as well. But that’s a few months ahead.
**IB**: Mm.
**Inf5**: I’m actually spending parts of this week on the thrombolysis process as a whole. But we have a sort of “side” routine, there is a routine for it.
[silence, bad network]
**IB**: Hm, let’s see here.
**Inf5**: … so we can say. Eh, therefore becomes…
**IB**: Now – now I lost it. Sorry, I lost the connection a bit.
**Inf5**: Yes. It says I have a weak connection.
**IB**: Okay. But you were saying, you have a routine…?
**Inf5**: There is a routine, meaning there is, let’s call it a backup routine. A system that we connect to our usual routine to handle “in-house” thrombolysis. Eh, the problem is just as you said, what happens in a stroke ward usually works well. There we have experienced nurses, they follow NIH [stroke scale] on the patients. TIA patients who have a stroke while in the hospital usually flow through well. Eh, post-operative orthopedic patients are a whole different matter.
**IB**: Yes?
**Inf5**: And that is one of the problems with the routines there. So, since the wards are different, the routines also vary a bit. It’s difficult to have the same routine for the entire hospital. They are similar, but who exactly handles what is different. So you could say that a stroke that happens during the day in a stroke ward, it’s naturally the senior physician there who handles it, unless they actively hand it over to the on-call doctor. What happens in an orthopedic ward, the medical on-call doctor will handle. It won’t, of course, be the orthopedic senior doctor managing a thrombolysis then.
**IB**: No, exactly.
**Inf5**: Eh, what we lack from the wards is the actual alarm. The patient who comes into the ER, we pull a stroke alarm. So radiology is alerted, the on-call doctor is alerted, ICU where we run the thrombolysis is alerted that something is on its way in. We don’t have that alarm from the wards. Practically speaking – and additionally, the ER staff are used to managing the Actilyse pumps that we routinely set up on-site at radiology.
**IB**: Mm.
**Inf5**: So, on the wards, there’s essentially an extra page in our routine, which involves – usually, it’s a nurse who sounds the alarm for a patient’s deterioration.
**IB**: Mm.
**Inf5**: On medical wards, they notify the on-call doctor. Or on the medical wards, they activate the senior physician. Usually, the nurse nervously speaks to a junior doctor first. So practically speaking, we never escape the fact that a junior doctor goes and looks at the patient, then realizes they should alarm the senior doctor. On the orthopedic ward, it will be an orthopedic doctor who checks the patient and then alarms the medical on-call doctor.
**IB**: Mm.
**Inf5**: Unfortunately, there have been a few cases where they haven’t done that, but have carefully ordered a CT brain in peace and quiet and waited for two hours. It’s not that such things don’t happen. But they should be aware of the routine, eh. It’s difficult to reach all the hospital’s doctors with how the routine works. But in an ideal world, you would in that situation eh, alarm the on-call doctor to the scene. The on-call doctor then makes the first assessments, and a junior doctor on-site, or if it’s the middle of the night, then it’s the on-call doctor – no junior doctor on duty – who writes the CT order that should trigger radiology. We have a specific stroke alarm CT that should flag for radiology. Practically speaking, this doesn’t always work, meaning when we’re coming from the wards where we can’t trigger the alarm, we also send this special CT order that should flag for radiology, and then we call down to the lead nurse in the ER because they should meet us at radiology with the thrombolysis cart.
**IB**: Mm.
**Inf5**: Where Actilyse is packed, ready to be mixed. Eh… and then the ER nurse should trigger the alarm, so radiology is alerted, and ICU is alerted.
**IB**: Exactly.
**Inf5**: And then everyone meets up at radiology. And at radiology, just like with other thrombolysis alarms, we follow the routine of… we send the order, place a green IV in the right arm, and then we go to radiology. Radiology finishes the scan they have on the table but doesn’t take in a new patient until we show up. If it’s a long wait, sometimes we wait a few minutes outside, or if there’s a complicated transfer, like the entire ICU being there with a patient. But usually, they’re standing there pretty impatiently waiting for us because it takes longer to get that green IV in place.
**IB**: Mm.
**Inf5**: And on a ward, all of that can take an unreasonable amount of time. Eh, ah…
**IB**: Mm. And just so I understand you correctly, someone notices the patient on a ward, often a junior doctor gets involved as an intermediary, and the medical on-call doctor or the senior doctor on the ward is eventually contacted…
**Inf5**: Yes.
**IB**: And that person then, if I understand correctly, once it's determined that this could require urgent treatment…
**Inf5**: Yes.
**IB**: Then they call the lead nurse in the ER, who in turn pulls the, presses the button, so to speak, so the regular alarm is triggered.
**Inf5**: Yes, so the regular alarm is triggered, and then they take the thrombolysis cart and go to radiology to wait there.
**IB**: Exactly.
**Inf5**: Because those nurses are the ones experienced in mixing and setting up the pump.
**IB**: Exactly. And if… if it’s a medical ward and there’s a medical senior doctor nearby, now I assume that person would perhaps go and look at the patient first before deciding…
**Inf5**: Yes, they would go and look at the patient, and the nurse or junior doctor should have pulled out the protocol, the thrombolysis folder.
**IB**: Mm.
**Inf5**: The stroke alarm folder, which we are renaming now. It used to be called the thrombolysis folder, but it’s being renamed to stroke alarm.
**IB**: Exactly.
**Inf5**: And the junior doctor on-site should start going through the contraindications there.
**IB**: And if the incident happens on another ward in the hospital? In surgery, orthopedics, gynecology, or wherever… eh, does the medical on-call doctor go there and check the patient first?
**Inf5**: Yes.
**IB**: That’s how it usually works?
**Inf5**: That’s how it works. Then the medical on-call doctor comes, and some doctor there should have printed out the protocol because it’s good to have the protocol in hand. Eh, I’ve never seen an orthopedic senior doctor start going through the contraindications; instead, we do that when we arrive on-site and assess the patient.
**IB**: Mm.
**Inf5**: The general rule we have is standard: if the patient has symptoms that could be stroke symptoms and they’ve occurred within, well, now within the last day for a thrombectomy alarm, and we still manage those the same way… So newly occurring stroke symptoms within the last day means we follow the protocol, basically. So that’s the first consideration one makes.
**IB**: When – when the decision is made, who takes the patient to radiology?
**Inf5**: Eh, the senior doctor or on-call doctor accompanies them. There’s usually some staff from the ward helping to push the bed.
**IB**: Mm.
**Inf5**: The nurse from the ER who meets them becomes the responsible nurse, so there might be an assistant nurse helping to push the bed. The on-call doctor or responsible senior doctor goes to radiology with them, as that’s where we make the thrombolysis decision.
**IB**: And it sounds like during the day at least, it’s always a specialist in internal medicine or neurology?
**Inf5**: Yes, it’s internal medicine. I’m the only neurologist, so it does happen that it’s me, but it’s internal medicine doctors who handle this, fundamentally.
**IB**: Okay. And what about during on-call hours?
**Inf5**: During on-call hours, it’s the on-call doctor, and we have an in-house on-call specialist doctor.
**IB**: So when the decision is made, it’s always the right competence level on-site, so to speak. It’s not an AT or ST doctor alone who’s responsible…
**Inf5**: No. We’ve said that since we have so few thrombolysis cases anyway, or I mean, say we have 3-4 stroke alarms a week and 1-2 that are relevant for intervention in a month. Eh, we’ve chosen not to have the emergency doctors handle it. We do have emergency doctors in the ER, but even there, the on-call doctor comes down to the alarms because it’s… yeah, the competence must exist within a certain group, and if we spread it out to the emergency doctors too, it would get even more diluted.
**IB**: Mm.
**Inf5**: And these alarms are the ones most of my colleagues feel the least confident about, that’s just how it is.
**IB**: Exactly. You touched a bit on the fact that sometimes a CT is ordered, and they wait, and maybe there isn’t full awareness of this in the hospital.
**Inf5**: Exactly.
**IB**: Could you tell me a little more about that?
**Inf5**: Yes. And as I said, what happens on a stroke ward usually works fine. What happens on orthopedic and surgical wards, we haven’t always reached them. Eh, what often makes it work is that there’s a junior doctor there, and junior doctors have recently enough been on medicine wards to at least call the medical on-call doctor and ask, “what should I do now?”
**IB**: Exactly.
**Inf5**: Otherwise, there’s this… you know, a small hospital where everyone is used to doing everything, and they think it’s just as well to check for a bleed before speaking to the medical doctor because they’ll want a CT anyway.
**IB**: Mm.
**Inf5**: And then suddenly you get a call from a nurse about a consult on a medical patient, and we’ve done a CT that shows nothing, but the patient is still paralyzed on one side.
**IB**: Mm.
**Inf5**: Yes, so things like that do happen. It’s gotten better, but eh… the biggest risk with thrombolysis alarms is that people try to be too competent. Everyone wants to make sure everything is done, the junior doctor wants to be absolutely sure it’s a stroke before calling the scary senior doctor. The issue is that everyone wants to be certain… it’s a problem, and we work quite a bit on trying to… yeah, just alarm, the worst that can happen is that someone like me comes running and says, “no, this isn’t for me, I’ll leave now.”
**IB**: Mm.
**Inf5**: Eh, but there’s this… yes, little hesitation to trigger an alarm for a patient who seems relatively stable. And then you think, “well, he is 82, and he was quite tired yesterday too, is it really something now, or will someone just think I’m being silly?”
**IB**: Mm.
**Inf5**: So yes, there’s that.
**IB**: Edu…
**Inf5**: And it’s always harder to pick up the pace. For example, if a nurse comes and… I mean, an experienced orthopedic nurse might not know stroke, and says, “he seems quite lethargic, he’s not even talking, and… he doesn’t seem to have the strength to lift his glass. Could you take a look at him when you have time?”
**IB**: Mm.
**Inf5**: And then you don’t quite pick up on it, and you don’t go there, and then you think, “but there’s something not right,” but you’re not sure, and then you talk to a third person, so there can be some delays there.
**IB**: Mm.
**Inf5**: It’s easier when someone comes in with a full ambulance as a clear stroke alarm, and you continue working from a high level; it’s harder to pick up the pace.
**IB**: Mm. Interesting point you raise! Do you provide any training? I’m thinking, especially for nurses and assistant nurses, who are the ones who see the patients the most.
**Inf5**: I think that’s a great question! Yes, we do train, and we have tried to train. Eh, as you may know, we’ve had this cursed pandemic for two years [laughs]. I don’t know how many training sessions have been canceled two weeks before because now we have to eh… now we can’t release people for training on this Tuesday afternoon…
**IB**: Mm.
**Inf5**: So that’s been… quite thin the past two years in that regard.
**IB**: Mm.
**Inf5**: I think we’ve had maybe one or two training sessions eh… the internal stroke training, the stroke training for stroke staff, has been canceled three times. The ones that run a bit longer, we’ve just started the fourth, and the first two were canceled. Eh, yes. But in principle, everyone agrees that we can’t keep canceling training, but it’s been – it’s been hard to push through.
**IB**: Mm, mm.
**Inf5**: And then these training sessions come last. We have a stroke nurse with the task of training the hospital, the staff at the hospital, and she’s been by the wards and given training during their… afternoon training sessions at almost all the wards. About swallowing checks after stroke and about acute management when a patient deteriorates. And she’s done nurse-to-nurse training.
**IB**: Mm.
**Inf5**: But, again, in the last two years, it hasn’t happened. They’ve canceled the internal training.
**IB**: I understand… Question, I just thought of something while we were talking about the care chain earlier, but… once at radiology, did I understand correctly that someone goes up with a cart with Actilyse?
**Inf5**: Yes, it’s a physical cart [laughs].
**IB**: Yes, and then… do you start the bolus in radiology, or is it done elsewhere?
**Inf5**: Eh, you start… in the best case scenario, the patient is already in place on the radiology table, and if we know that… let’s say that the on-call doctor feels confident that we’re going to administer Actilyse unless we see a bleed, that radiology isn’t a contraindication. This assumes that the on-call doctor assessing the patient between times does a first assessment and then goes through the contraindication list in the hallway on the way to radiology, reviews the patient’s record on the computer at radiology but feels confident that we’ll give it unless there’s something unexpected.
**IB**: Mm.
**Inf5**: Then they tell the nurse to start mixing. If we get in between the CT and the angio, then we give… go in and administer the bolus and set the pump, make a short pause for the contrast agent, and then continue. Quite often, we don’t make it…
**IB**: Mm.
**Inf5**: And then we go in after the angio and set it basically before we transfer the patient back to the bed.
**IB**: Exactly.
**Inf5**: The Actilyse itself is given in our ICU, because that’s the only place where we have enough staff to do frequent checks. That is also under review; it would be better if it were done in the stroke ward… but it’s a staffing issue.
**IB**: Exactly… O – overall, would you say… now you’re speaking for the whole hospital, but, like… do you think that the routine you have works well? Or is there something you think works less well?
**Inf5**: Mm. Eh… I think it works okay.
**IB**: Mm.
**Inf5**: We need to revise it in various ways. What we’re working on now is reviewing the possibility of… the ambulance driving directly to radiology and not going through the ER as we used to do.
**IB**: Mm.
**Inf5**: I think there’s quite a lot to gain from that. The idea is that we have quite good routines. The enormous difficulty of maintaining continuous staff training so that we follow our routines is a challenge!
**IB**: Mm.
**Inf5**: There are new nurses starting all the time; there’s a lot of… everyone trying to be competent. If the on-call doctor isn’t alert in the ER room, you might get patients where they both insert catheters, take EKGs, and do the most astonishing stabilization efforts for the patient, because everyone wants to be competent.
**IB**: Mm.
**Inf5**: Eh, it’s… the stroke alarms are quite different from the other alarms…
**IB**: If – if you think specifically about those who fall ill in the hospital?
**Inf5**: What did you say?
**IB**: If you think about those patients who fall ill in the hospital?
**Inf5**: Exactly, for those who fall ill in the hospital, we have fewer problems with well-meaning ER nurses. But to be honest, it’s much harder to get that green IV in place. The ER nurses are used to quickly getting an IV in.
**IB**: Mm.
**Inf5**: On the wards, you can end up standing and waiting while someone sticks and sticks again… and, there, we don’t have a clear routine other than a competent on-call doctor eventually stepping in and saying, “We’re going to radiology, and the ER nurse can help with the IV there.”
**IB**: Mm.
**Inf5**: “We can’t do more here and now.” But that’s… so it’s a different type of problem on the wards.
**IB**: But – but overall, from the time someone calls you and says, “I think this patient has had a stroke,” to the time you’re standing there in radiology, is there anything in that chain where you see time could be cut or where things could run smoother?
**Inf5**: Mm. You could say if… eh, if the experience on the wards was greater, you could, of course… but this is where it varies by ward, it goes faster on a medical ward. There’s a doctor on-site who can drive the issue… take the patient to radiology immediately and meet up with the on-call doctor at radiology and hand over there.
**IB**: Mm.
**Inf5**: Eh, in the best case scenario, even orthopedics could jog down with the patient, and we’d meet the patient at radiology… in practice, that’s not going to work.
**IB**: Mm.
**Inf5**: It requires someone to be there and make things happen.
**IB**: Mm.
**Inf5**: If I… just from experience, if I tell the orthopedic ward, “Take the patient to radiology right away, I’ll write the order from here based on what you’ve described and what I see in the record, and we’ll meet there,” the worst that happens is that we CT the head one too many times on an 82-year-old man, and it solves itself.
**IB**: Mm.
**Inf5**: But then I’ll end up standing at radiology for 20 minutes waiting… eh, because there may very well be someone who’s called a transporter…
**IB**: Yes.
**Inf5**: And is waiting for transport to arrive.
**IB**: Exactly.
**Inf5**: That’s where we save time when an experienced medical on-call doctor comes and picks up the pace. Because that’s what you do when you arrive.
**IB**: Mm… I see. And…
**Inf5**: So, I can see that if we got routines working differently, you could save time, but in practice, I’m doubtful that it would work. And it’s better to have routines that work.
**IB**: Have you received any, I mean… feedback from any colleague, or anyone else in the staff who has been involved in a case like this before? Someone who perhaps felt it didn’t go so well and had suggestions on how it should be done instead?
**Inf5**: Oh, yes! We get that all the time [laughs].
**IB**: Yeah, is there anything you can share with me?
**Inf5**: Yes, well… we talk through these things usually afterward. Especially those that happen in-house… ehm, there’s also a… I don’t know… a feedback loop to people, everyone feels a bit bad about being involved in these things.
**IB**: Mm.
**Inf5**: We go through, “I checked him first and thought something was wrong, but didn’t react until I went back with the lunch.” I mean, people need to talk things through afterward.
**IB**: Mm.
**Inf5**: Eh… so yes, pretty much after every thrombolysis alarm or stroke alarm, people… eh, say that didn’t go so well. There are always little things that don’t go right… ehm, this… what shall we call it, we call it the thrombolysis folder, but… the stroke alarm routine that’s been printed out. When they print it out and it comes out on another printer in the hospital. Everyone goes around looking for it. No one starts anything. I mean, this stressful situation can arise with all alarms.
**IB**: Mm.
**Inf5**: So, as I said, the routine… works if you follow it. But there are often things that don’t go smoothly, there’s a lot to review in the whole thing.
**IB**: Mm. And thinking specifically about that group who fall ill while in the hospital, what do you think… which step in the care chain do you think is, let’s say… the most likely to cause delays, or…
**Inf5**: Really, the first step.
**IB**: Mm, detecting it?
**Inf5**: Detecting it and trusting what you’ve detected. And being willing to act on it. It requires steps: an assistant nurse tells a nurse who goes and looks at the patient, feels a bit uncomfortable, the doctor is at lunch, should we disturb him for this? Then they decide to disturb him for this. Then tell the doctor, who’s having lunch, and the doctor needs to feel that we need to act quickly on this.
**IB**: Mm.
**Inf5**: A ehm… yeah. An orthopedic senior doctor my age hasn’t always been involved enough to realize that there’s a different pace for stroke patients, to be honest.
**IB**: Mm.
**Inf5**: They go and look at the patient, then realize that we can’t handle this ourselves, there’s a whole alarm system. Eh, much of that information needs to be spread, but just the step before you’ve picked up the pace is the hard part.
**IB**: Mm.
**Inf5**: Eh… once you have a medical on-call doctor there, with a protocol in hand, everything flows after that. Then it’s, I almost said minor issues, but it’s about getting an IV or not getting an IV. That’s what limits the speed on the ward.
**IB**: Mm.
**Inf5**: But the step before that, where… and it’s just training efforts, you just have to go through it. If you’ve heard about it a few times, you react to it, then you have a strategy and a plan. And everything goes a little faster if you feel confident that “this is what we do here.” “Right, we have a routine here, so let’s follow it and do as it says.”
**IB**: Mm.
**Inf5**: Then it works. But, so to speak, the step before starting the routine creates delays in “in-house” cases.
**IB**: Yes, but it’s fascinating to hear how it works at your place. Eh… is there anything else you want to add that you feel we haven’t discussed? I feel I’ve gotten quite a lot of information.
**Inf5**: Yes, I mean, you could say that the later steps are exactly the same. Once we’re at radiology, everyone who needs to be there is there, so we just do what we usually do.
**IB**: Mm.
**Inf5**: So it’s the time before that which distinguishes “in-house” from the others.
**IB**: And if an intervention becomes relevant, and so on, then it’s from ICU that you call? It has to be [university hospital] that does them?
**Inf5**: Yes, [university hospital] does them for us, and it’s the same system there. We sometimes have… here, it can be a bit of an issue with the ER sometimes, though not too often. Because practically speaking, if Actilyse isn’t relevant, but only thrombectomy, then the patient should go from radiology to the ER because it’s much faster to arrange transport from there.
**IB**: Mm.
**Inf5**: But then there are a number of patients who are admitted to a ward who end up in the ER for half an hour or an hour while they are calling back and forth. Then maybe there’s no thrombectomy, and the patient should go back to some ward. And then there’s some back-and-forth about whether the patient should go back to the orthopedic ward or if the patient should be in the stroke ward now, and… on an overloaded ER, this extra patient who doesn’t really belong there and maybe shouldn’t be on the emergency list, so we need… we also need to have a clear routine so people know what’s what.
**IB**: Exactly.
**Inf5**: Because there can be a lot of back-and-forth because no one has really thought the situation through.
**IB**: Mm.
**Inf5**: And someone like me isn’t aware that we have a problem, because the patient can’t still be registered when you’re supposed to check them out from the emergency list, otherwise the system doesn’t work. And patients who aren’t on the emergency list, the ER nurses don’t feel they’re responsible for, so who owns the patient? I mean, there’s… as I said, it’s those kinds of things we need to go through now.
**IB**: Mm, mm.
**Inf5**: So there’s also a difference between those who come in through the ER, where there’s no Actilyse, and those who go back to the ER, there aren’t those kinds of administrative problems that as a doctor, I never think about, but they just get in the way because everyone asks me if they should check the patient out from the ward and into the emergency list.
**IB**: Yes…
**Inf5**: Yes. And it’s, yeah…
**IB**: Organization… it can be difficult sometimes…
**Inf5**: Exactly. So even there, we just need to decide how we handle it, and then everyone will be happy. If everyone knows what’s expected, there’s less back-and-forth, and that just takes time and energy from people…
**IB**: Yes, yes…
**Inf5**: Yes.
**IB**: I forgot to ask you just at the beginning. I understood that you’re a neurologist.
**Inf5**: I’m a neurologist and internal medicine specialist.
**IB**: And internal medicine specialist, yes. And how long have you been responsible for the stroke chain?
**Inf5**: Eh, officially, I’ve been responsible since I became a double specialist, which was in 2010.
**IB**: Yes. Then I don’t think I have any more questions.
**Inf5**: No.
**IB**: It was really interesting to hear!
