# Supplementary material for: Exploring strategies for management of in-hospital stroke in Sweden: A qualitative study
Source: PLoS One. 2024 Nov 26;19(11):e0313765. doi: 10.1371/journal.pone.0313765 (PMC11594569; doi:10.1371/journal.pone.0313765)
Supplement: S6 Text — (DOCX) [file pone.0313765.s007.docx]

**IB:** You can start by telling me a little about your background! You're a specialist in…?
**Inf6:** I'm a specialist in internal medicine since 2007, and also in emergency medicine since 2014. I've worked at [hospital] since I finished my training, and I did my AT (internship) here as well. I started in 2000, and…
**IB:** Mm.
**Inf6:** Now I work half-time in medicine and half-time in the ER, 4 weeks each in rotation throughout the year… ehm, yes. And a few years ago, our stroke doctor retired, so I was asked if I wanted to take over responsibility for the stroke ward, and after a bit of consideration, I said yes. Since then, I’ve mostly been at the stroke ward when I’m on my medicine placement.
**IB:** Okay, which year was that?
**Inf6:** Well, time flies. It was probably 5 years ago, if not even longer.
**IB:** Mm, okay. My first question is… at [hospital], is there a written routine for what to do if a patient is suspected to have a stroke, like an in-house patient suspected of having a stroke?
**Inf6:** Mm, yes, there is. I actually brought it out just before this interview.
**IB:** Okay, yes, feel free to send it to me after we finish talking.
**Inf6:** Yep!
**IB:** But can you explain a little, go through the different steps? What does the routine say?
**Inf6:** Yes, we have a routine for the nurses on the ward and also for the ward doctor or the primary on-call doctor if it’s after hours. If we start with the doctor's routine, ehm… it says that the doctor should assess whether the patient is eligible for a "Rädda hjärnan" (Save the Brain) alarm, write a referral for a CT scan, start filling in the indications and contraindications for thrombolysis and the NIH stroke scale, and follow the patient to radiology. Then make the thrombolysis decision in consultation with the senior on-call doctor. There's also a section for the internal medicine senior on-call doctor that says they should join the radiology department at that point.
**IB:** Mm.
**Inf6:** And for the nurses’ routine, it says they should contact the responsible ward doctor or the on-call doctor at the ER if it’s after hours. There’s a form they need to fill in with the criteria for a “Save the Brain” alarm, the same as what the ambulance uses. They also need to bring out the thrombolysis folder and notify radiology, and so on…
**IB:** Mm. And do you feel that… how does it work in practice? Do you feel like people follow the routine?
**Inf6:** I can say that we don’t have in-house “Save the Brain” alarms very often. This past summer, we actually had one where I was involved. It wasn’t on our usual stroke ward, but on another medical ward, and there were some uncertainties. They didn’t know… we have a thrombolysis folder, but they couldn’t find it… or they didn’t have one, so we had to get one from the neighboring ward. So it didn’t go so smoothly there. However, if it had been on our stroke ward, I believe it would have gone much better because they’re more used to it… more familiar with the patients and the whole process.
**IB:** Mm. And you have other departments, right? And an operating department?
**Inf6:** Yes, and we also have the ICU and an orthopedic department.
**IB:** How do you think the awareness of the routine is in those departments?
**Inf6:** The orthopedic department used to be under the medical clinic, but about a year ago, it switched to the orthopedics department, so I don’t really know… how it is now. When it was still under the medical clinic… it was about the same as the other medical wards. There wasn’t great knowledge or familiarity with the routine. Now, I’m not sure how things are there… I must say, there’s a lot of new staff there too. So there’s a risk that they’re not fully updated, perhaps.
**IB:** Mm, no… but if we walk through the care chain, let’s say… someone notices or suspects that an in-house patient has had a stroke. It’s usually someone from the ward staff, perhaps a nursing assistant or a nurse.
**Inf6:** Yes?
**IB:** What does that person do then?
**Inf6:** Ehm… a nursing assistant would probably go talk to the nurse. If it’s the nurse, they would contact the ward doctor.
**IB:** Mm. And then what does the doctor do?
**Inf6:** Then they go and check on the patient, make an assessment. Is this someone who’s eligible for a “Save the Brain” alarm, and if so, activate the alarm.
**IB:** Mm. How is the alarm activated?
**Inf6:** Well, you tell the nurse that it’s a “Save the Brain” alarm, and for an emergency CT, they have in their routine that they call radiology and so on.
**IB:** Yeah… I’m wondering, is there like a physical alarm button somewhere that triggers everyone’s pager?
**Inf6:** No, no, there isn’t.
**IB:** There’s no pager code for “Save the Brain” in the hospital?
**Inf6:** No, there isn’t.
**IB:** Not even when an ambulance comes to the hospital with a suspected stroke patient?
**Inf6:** No, then they call the primary on-call doctor in the ER.
**IB:** Okay. So, it’s more of a situation where they determine it’s an emergency, and the nurse calls radiology to let them know you’re coming?
**Inf6:** Yes, exactly, and they also call the internal medicine senior on-call doctor, who should be included as well.
**IB:** And if this happened in the ICU or orthopedics, for example, who would the nurse contact?
**Inf6:** They would either call the internal medicine senior on-call doctor or, most likely, the primary on-call doctor in the ER, I think.
**IB:** And that person would then go and check on the patient?
**Inf6:** Yes, exactly.
**IB:** Yeah… could you say that there’s a limited number of people who can make the decision to sound the alarm? Or could a ward doctor in orthopedics also decide that it should be a stroke alarm?
**Inf6:** Yes, they can do that…
**IB:** And in practice, does that happen?
**Inf6:** The orthopedic doctors we have are fairly “all-round,” so I think they would feel confident enough to trigger such an alarm, yes.
**IB:** Mm. Okay. From the point where the alarm is triggered, what happens then?
**Inf6:** Then the staff, the nurse, should look at the protocol or routine and start making the necessary calls. The doctor who’s managing the patient should write the referral to radiology and start examining the patient, doing the NIH stroke scale, and thinking about indications and contraindications, and so on.
**IB:** Mm… and then you go to radiology?
**Inf6:** Mm. As soon as you can… it happens so rarely from the wards. But the idea is that you should start transporting the patient to radiology immediately, and during that time, on the way, ehm… you perform the NIH stroke scale and so on. And if you haven’t finished by the time you get to radiology, you continue there if needed.
**IB:** Exactly. And… once at radiology, who are the people present there?
**Inf6:** It’s the nurse and nursing assistant from the ward, the doctor who’s with the patient, and then the ICU nurse also comes because they’re the ones who mix and administer thrombolysis, or Actilyse. And the internal medicine senior on-call doctor should be there too, hopefully, they should be there.
**IB:** Mm… so… then the patient is on the table, you do a CT brain scan, and if there are no contraindications, the patient should receive thrombolysis. Do you start treatment right there at radiology? Or how do you do it at your hospital?
**Inf6:** Yes, we start right there at radiology, eh… and often, they also get a CT angiography during the ongoing Actilyse treatment.
**IB:** Exactly… precisely… and after radiology, where does the patient go?
**Inf6:** Then they go to the ICU, where they stay for 1-2 hours during the thrombolysis treatment, and maybe a little longer afterward. Usually, it’s no more than two hours.
**IB:** Mm. And if an intervention is needed?
**Inf6:** Then they go directly from the ICU to [university hospital].
**IB:** Exactly… yeah… and how do you feel the care chain works at your hospital?
**Inf6:** Ehm… when patients come from outside, like through the ER, it usually works very well. Eh, from the wards, like I said, I had one case this summer… and it was a bit hesitant because they couldn’t find all the papers and such, but once we got to radiology… everything went smoothly as usual, which is good.
**IB:** Mm… one possible step… or let me ask you first, what do you think could be the limiting step in the care chain? If we think about making it faster, where do you think there’s the most time to save?
**Inf6:** If it’s another ward than the stroke ward, then I think it’s about… the person who notices the patient’s unusual symptoms having the confidence to act on it… and quickly too… I mean that they dare to call the doctor right away and not ponder over it for a while.
**IB:** Yes… do you think that happens?
**Inf6:** That they ponder?
**IB:** Mhm.
**Inf6:** Ehm… yes, I felt this summer that they had been pondering for a while before I was contacted.
**IB:** Yes… and could that be avoided in some way? What do you think could be done to improve that?
**Inf6:** Well, you could remind people of the routine now and then. After this happened in the summer, we made it so that the “Save the Brain” routine for the wards became the routine of the month the following month, so that everyone was reminded and discussed it in staff meetings, and everyone had to read through it, and so on.
**IB:** Exactly, so some kind of training element for the whole hospital?
**Inf6:** Yes, exactly.
**IB:** And in the years you’ve been responsible for this, has anyone from the staff suggested improvements, or given feedback on what has worked well or not so well? Anything you’d like to share?
**Inf6:** Regarding the “Save the Brain” routine?
**IB:** Yes, for in-house patients.
**Inf6:** Not that I can recall… not that specific routine. However, our ward nurses and stroke nurses are very good at suggesting improvements to other stroke routines… but I don’t remember anything specific about the “Save the Brain” routine.
**IB:** Mm… but just so I understand correctly. Someone in the staff suspects that a patient has had a stroke on a ward… and that person, if it’s a medical ward or the stroke ward, they call the doctor on the ward? Is that correct?
**Inf6:** Yes, that’s right.
**IB:** And if it’s another department in the hospital, they probably also contact their ward doctor, but maybe also the internal medicine senior on-call doctor?
**Inf6:** Well, we only have the orthopedic ward here, and from what I know, their orthopedic doctors are usually in surgery when they’re not on the ward, so they usually call down to the ER, to the primary on-call doctor.
**IB:** Okay.
**Inf6:** We have a non-specialty specific ER, so the primary on-call doctor handles all patients.
**IB:** Exactly. So would you say it’s part of the primary on-call doctor’s duties to go up to the wards and make an assessment?
**Inf6:** When it comes to “Save the Brain,” yes, that’s the case.
**IB:** Yes… so then a doctor would come up to make an emergency assessment of the patient?
**Inf6:** Yes.
**IB:** And they go through NIHSS and the indications and contraindications for thrombolysis or thrombectomy.
**Inf6:** Mm.
**IB:** Then they decide it’s a stroke alarm, and according to the routine, the nurse is responsible for initiating the alarm?
**Inf6:** Yes, exactly.
**IB:** And the doctor writes the referral to radiology, and then you go to radiology…
**Inf6:** Mm.
**IB:** Let’s say it’s a nurse and perhaps a ward doctor, and since there’s no physical alarm that goes off on a pager, who notifies the ICU nurse to come to radiology? And who calls the internal medicine senior on-call doctor?
**Inf6:** The nurse does that, according to the routine.
**IB:** Okay. So then everyone meets at radiology, the patient gets a CT scan. If thrombolysis is needed, it’s administered right there, no delays at radiology?
**Inf6:** No.
**IB:** Then the patient gets a CT angiography, goes to the ICU… and if there’s an indication for thrombectomy, the patient is transferred from the ICU to [university hospital], so transport is arranged…
**Inf6:** Yes.
**IB:** Is it then the internal medicine senior on-call doctor who takes over and handles that?
**Inf6:** Yes, the senior on-call doctor is responsible for that. The senior doctor handles ICU and coronary care unit patients too, so it automatically ends up on their plate.
**IB:** Mm. Just a question about delays in the first step, detecting the stroke… you’ve never been involved in a case where there was a significant delay? Where maybe they waited for hours?
**Inf6:** No, I’ve never heard of that happening.
**IB:** Okay… but you still think that some form of training initiative from time to time would be helpful so that the hospital staff feels comfortable reacting?
**Inf6:** Yes, absolutely, and especially for those who don’t work with stroke patients regularly, they might need a bit of a reminder because it’s easy to forget things you don’t deal with every day.
**IB:** Of course… alright, is there anything else you want to add to what we’ve discussed?
**Inf6:** Eh… no, not really, not that I can think of at least.
**IB:** It sounds pretty “straightforward” so to speak…
**Inf6:** [silence]
**IB:** Then I think we can wrap up here.
